# The Temperature Sensitivity of Soil CO_2_ Release Is Modified by Virus–Bacteria Interactions

**DOI:** 10.34133/research.1479

**Published:** 2026-11-13

**Authors:** Jianming Xu, Di Tong, Jiachun Shi, Junjie Xie, Nan Zhang, Zhongmin Dai, Shuai Zhang, Randy A. Dahlgren, Bin Ma

**Affiliations:** ^1^ State Key Laboratory of Soil Pollution Control and Safety, College of Environmental and Resource Sciences, Zhejiang University, Hangzhou, Zhejiang, China.; ^2^ Zhejiang Provincial Key Laboratory of Agricultural Resources and Environment, Zhejiang University, Hangzhou, Zhejiang, China.; ^3^ The Rural Development Academy, Zhejiang University, Hangzhou, Zhejiang, China.; ^4^ Department of Land, Air and Water Resources, University of California, Davis, CA, USA.

## Abstract

The temperature sensitivity (*Q*_10_) of soil carbon (C) release is key to predicting C-climate feedback, yet omitting microbial interactions, especially with soil viruses, introduces substantial uncertainty. To address this, we performed 30- and 365-d incubations with metagenomics and metatranscriptomics profiling. We found that soil viruses markedly decreased *Q*_10_ during 30-d incubation. Over 365-d incubation, however, their effects on microbial metabolism and *Q*_10_ were temperature-dependent. The relative abundance of pathways related to viral replication, including those for lytic cycle initiation (e.g., propanoate metabolism decreased by 15.24%) and the energy supply (e.g., lipoic acid metabolism decreased by 20.95%), became less abundant as temperature rose from 15 to 35 °C. Metatranscriptomics data confirmed this trend, with up-regulation of metabolic functions related to viral infection and replication at 15 °C. Thus, temperature-driven variations in viral activity underlie the contrasting roles of soil viruses in regulating *Q*_10_. This study provides the first evidence of temperature-dependent viral regulation of microbial metabolism and *Q*_10_, highlighting the need to incorporate soil viruses into C-climate models.

## Introduction

Global warming exerts a positive feedback on the mineralization of soil organic carbon (SOC), which is projected to increase by ~40% by the end of this century [1]. The temperature sensitivity of soil C release (*Q*_10_, the factor describing the increased respiration rate with a 10 °C increase in temperature) is considered a universal parameter and has been intensively studied to project the magnitude of soil C release under climate warming [2–4]. However, uncertainties associated with the parameterization of *Q*_10_ severely limit its application in climate and biogeochemical models, even leading to erroneous results. This ensues from the myriad of factors affecting *Q*_10_, including the soil microbiome, substrate quantity/quality, mineral protection, biochemical recalcitrance of organic matter, and several other environmental factors (e.g., soil water and oxygen contents) [5,6]. Increasingly, studies highlight the dominant role of soil microbiomes in regulating *Q*_10_, whether on a global scale or in short- versus long-term soil incubation studies [7,8]. However, almost all existing studies investigating the control of *Q*_10_ by soil microbes have focused solely on soil microbial biomass, richness, and community composition. These studies neglect the complex interactions between microorganisms, which can substantially alter the temperature dependence of microbial respiration [9,10]. Currently, how interactions among soil microbial species influence *Q*_10_ remains unclear, let alone the effects of the more complex virus–microbe interactions.

Viruses are almost an order of magnitude more abundant than prokaryotes and can have a profound effect on Earth’s biogeochemical cycles [11,12]. It is estimated that viral lysis annually releases approximately 145 Gt of C, 27.6 Gt of nitrogen (N), and 4.6 Gt of phosphorus (P) into the marine environment [13]. Microbes have a high affinity for the multitude of products released during viral lysis, thereby recycling a portion of the nutrients and energy generated and preventing their utilization by higher trophic levels [14,15]. This process is known as the “viral shunt”, whereby lysate dissolved organic matter (DOM) is converted from a labile pool to a more recalcitrant pool [16]. Moreover, cell debris and other lytic products that are resistant to degradation can be aggregated and retained in the environment for prolonged time periods, the so-called “viral shuttle” process [17]. In soil systems, the viral shuttle also promotes the formation of mineral-associated C pumps (e.g., FeOC accumulation), driven by the strong affinity of Fe(hydr)oxides for cellular debris released through viral lysis [18–21]. While the full quantitative impact of viruses on soil biogeochemistry is not yet resolved, empirical evidence from natural environments confirms the existence of both the viral shunt and shuttle pathways in virus-mediated C cycling [22–24]. Both the quantity and the quality of soil organic matter (SOM) substrates and mineral protection are strong regulators of soil C release and *Q*_10_ [25–27], thereby suggesting that viruses may play an important role in C release and *Q*_10_ through the viral shunt and viral shuttle pathways.

Previous studies on soil viruses and C cycling have been performed under short-term incubations (weeks to months) at optimal growth temperature (around 25 to 30 °C) [22,28,29]. Yet, they universally overlook the temporal evolution of substrate quality over approximately 1 year and the altered virus–microbe interactions at temperatures above their optimal growth temperature (>30 °C) [9,30–32]. This narrow focus is a significant gap, as long-term incubations reveal that shifts in substrate availability and microbial traits exert a major influence on soil C release and *Q*_10_, factors that short-term studies cannot capture [7,33,34]. Moreover, viral infection capacity, virulence, and replication rate increase as temperature rises within their optimal range, but once the optimal temperature is exceeded, they decrease sharply [32,35]. Given the temperature-dependent properties of viral infections, we hypothesize that the impact of soil viruses on *Q*_10_ switches from inhibition to facilitation once the incubation temperature exceeds the optimal growth temperature (>30 °C). To bridge this knowledge gap and test our hypothesis, we employed a combined approach of metagenomics and metatranscriptomics on soil samples from 30- and 365-d incubations across a temperature gradient (15, 25, and 35 °C). The aims of this study were to (a) clarify the role of viruses in soil C release and *Q*_10_, (b) reveal how soil viruses affect soil C release and *Q*_10_ over different time scales and temperature ranges, and (c) identify the dominant drivers of viral-regulated *Q*_10_ and the related mechanisms at different temperatures.

## Results and Discussion

### Effects of soil viruses on *Q*_10_

To fully explore the effects of soil viruses on *Q*_10_ in the short and long term, incubations were performed over 30 and 365 d. For the short-term incubation, regardless of soil type, groups receiving active virus (V) significantly decreased *Q*_10_ of between −27.9% and −0.6% compared to no added virus (SM) (Fig. 1A and B and Fig. S3C and D). However, the viral effects on *Q*_10_ in the long-term incubation did not always show a consistent pattern (Fig. 1C and D). For example, while *Q*_10_ was significantly decreased under lower-temperature incubation (15 to 25 °C), it was significantly increased in the LN-C groups. Similarly, when *Q*_10_ increased in other groups under higher-temperature incubation (25 to 35 °C), it was suppressed in the LN-C group (Fig. 1C and D). In addition, more complex trends were found between *Q*_10_ in the DV and V treatments. The viral effect on *Q*_10_ in long-term incubations ranged from −7.1 to 42.7% in the higher temperature range and −32 to 14.8% in the lower temperature range, which was significantly higher than that found in the short-term incubation (Fig. 1 and Fig. S3). A novel and striking finding was the consistent reversal of the viral effects on *Q*_10_ between the 15 to 25 °C and 25 to 35 °C ranges during long-term incubation, observed universally across soils and experimental groups. This previously unreported pattern prompted a mechanistic investigation into key factors potentially responsible for the shift, such as DOM recalcitrance, mineral protection, nutrient availability, and microbiome dynamics, as detailed in prior work [36,37].

**Fig. 1.**
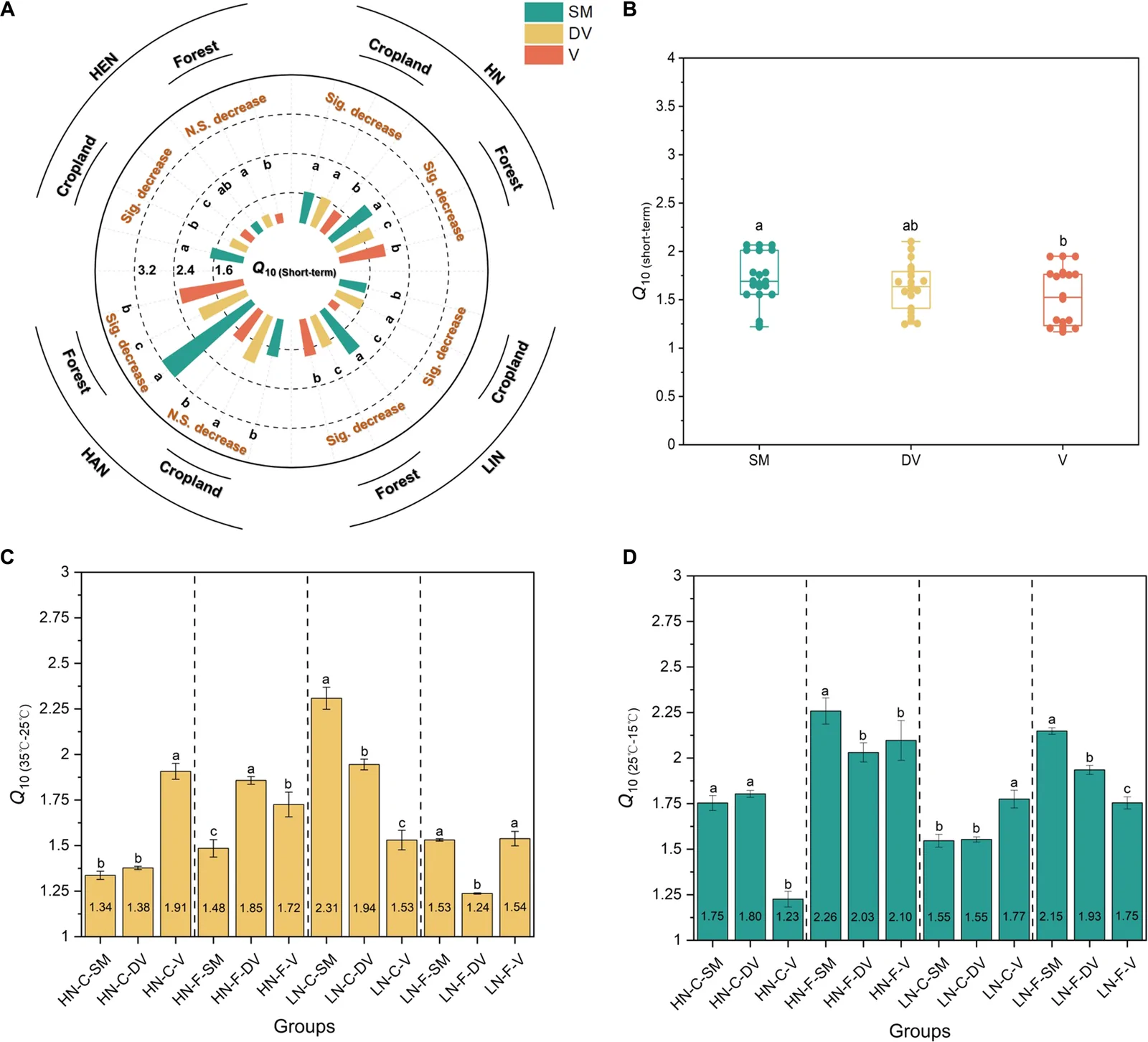
Variation in *Q*_10_ (mean ± SD) among treatments under short-term (30-d) (A and B) and long-term (365-d) incubation [(C), higher temperature range (25 to 35 °C); (D), lower temperature range (15 to 25 °C)]. SM, no added viruses; DV, inactivated viruses; V, active viruses; 35, 25, and 15 signify different temperatures (35, 25, and 15 °C) during the incubation. HN-C/LN-C/HAN-C/HEN-C and HN-F/LN-F/HAN-F/HEN-F represent soils from cropland and forest in Hunan, Liaoning, Hainan, and Henan provinces, China, respectively. Different lowercase letters represent significant differences at *P* < 0.05. The details of calculating *Q*_10_ in 30- and 365-d incubations can be found in the Supplementary Materials.

### Response of DOM components and FeOC to soil virus treatments

While a large fraction (50% to 80%) of lysate DOM, including bioavailable compounds such as amino acids and carbohydrates, is rapidly consumed and respired [16,38,39], the recalcitrant fraction of lysate DOM (indicated by SUVA254, SUVA260, and HIX) accumulates over successive cycles of microbial recycling [16,17,22,40,41]. Upon incubation, recalcitrant DOM components reflected by aromaticity degree (SUVA254), hydrophobic degree (SUVA260), and humification degree (HIX) all significantly increased with the addition of active soil virus, except for the LN-C groups (Fig. 2A). SUVA254 and SUVA260 in the HN-C and HN-F groups increased with viral lysis at all 3 temperatures over the 365 d of incubation, with the maximum effect as high as 52.5% (Fig. S4). In contrast, SUVA254 and SUVA260 in the LN-C groups decreased at all temperatures, whereas the LN-F groups increased at 15 °C, but decreased at warmer temperatures (25 and 35 °C). Except for the notable increase in SUVA254 and SUVA260 in the HN-F groups at 15 and 25 °C, these parameters all decreased with soil virus addition in the other groups (*P* < 0.05). HIX showed a consistent increase of 1% to 5% at 35 °C with the addition of active virus for all soil types (Fig. S4).

**Fig. 2.**
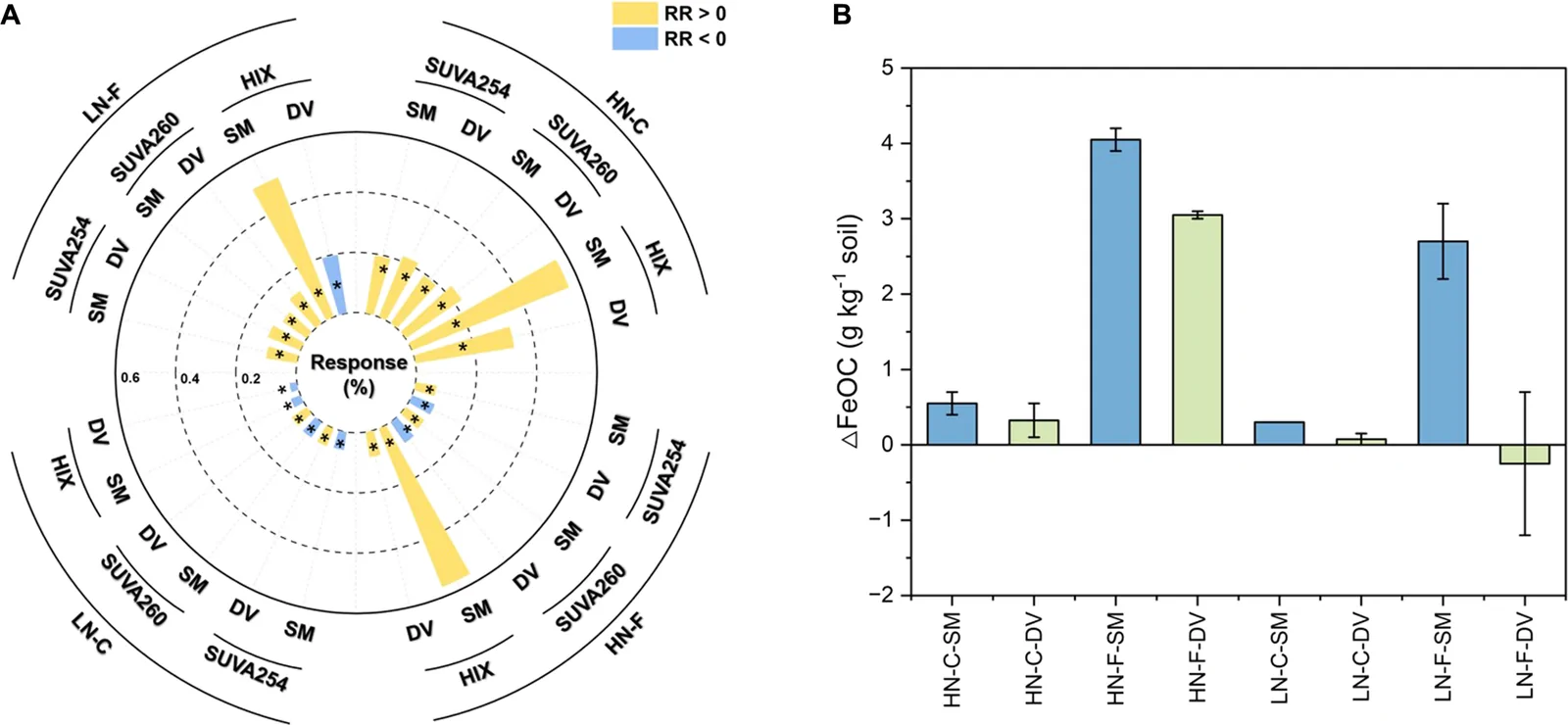
Response of DOM components and FeOC (mean ± SD) to soil virus treatments after the pre-incubation. (A) Changes in DOM components. An asterisk indicates a significant difference between the virus group and the CK groups (SM and DV). In each panel, the yellow and blue bars denote positive and negative numbers, respectively. The length of the bar represents the absolute value of the response ratio. HIX, SUVA254, and SUVA260 indicate the degree of humification, aromaticity, and hydrophobicity of DOM, respectively. (B) Difference in FeOC between virus groups (V) and CK groups (SM and DV). SM, no added viruses; DV, inactivated viruses; V, active viruses. HN-C/LN-C and HN-F/LN-F represent incubated soils from cropland and forest sampled in Hunan and Liaoning provinces, China, respectively.

When comparing DOM components between the DV and V groups, there was no clear trend as the DOM characteristics shifted among soil types and incubation temperatures (Fig. S4). In addition, these DOM indicators showed a significant positive relationship with soil C release (Figs. S7 to S9), inferring that recalcitrant DOM components accumulated due to the recycling of lysate DOM by the microbial community. SUVA254 and SUVA260 appeared to be more consistent indicators of the viral shuttle mechanism than HIX, which only showed a significant relationship with soil C release at 25 °C, whereas SUVA254 and SUVA260 showed significant correlations with C release across all incubation temperatures (Figs. S7 to S9).

Soil viruses stimulated the accumulation of FeOC (Fig. 2B and Fig. S5), which is resistant to degradation by soil microbes and plays a crucial role in SOC sequestration [20,42]. The content of FeOC was measured to assess whether more OC was protected by minerals due to viral lysis. Compared with the SM group, the addition of viruses significantly increased the content of FeOC regardless of incubation temperature or time, with the exception of the LN-F groups at 25 °C (Fig. 2B and Fig. S5). In contrast, the FeOC content was more variable in the DV groups, with changes occurring at different incubation temperatures and times. The formation of FeOC appears to result from the combined effects of C decomposition, microbial processing, and viral dynamics over the incubation period. However, no significant relationships were found between FeOC levels and the viral impact on soil C mineralization or *Q*_10_ (Fig. S6).

### Relationships of abiotic and biotic properties with virus-mediated *Q*_10_

Both the content and quality of soil nutrients can influence C release and *Q*_10_ (Notes S1 and S2). Nutrient content affects *Q*_10_ based on whether microbes gain sufficient energy and materials to offset the cost of producing the related enzymes [43]. Soils with lower soil C content requiring limited microbial investment result in lower soil C loss, whereas C-rich soils would have a higher C loss [2,8,27]. In contrast, the quality of soil nutrients affects *Q*_10_ owing to the activation energy required to release nutrients [2]. In general, more labile substrates require less activation energy, resulting in higher *Q*_10_ values [44]. While the relationships between measured nutrient and microbial properties and *Q*_10_ were highly consistent in the low-temperature regime [*Q*_10 (25-15)_] and for the short-term incubation [*Q*_10 (short-term)_], the corresponding correlations were either opposite or not significant for the high-temperature regime [*Q*_10 (35-25)_], shown in Fig. 3A. For recalcitrant DOM components, HIX was the only parameter positively correlated with *Q*_10 (35-25)_, whereas SUVA254 and SUVA260 were positively associated with *Q*_10 (35-25)_ and negatively related to *Q*_10 (25-15)_ and *Q*_10 (short-term)_. These results suggest that SUVA254 and SUVA260 are more sensitive indicators reflecting the effects of viruses on *Q*_10_ dynamics.

**Fig. 3.**
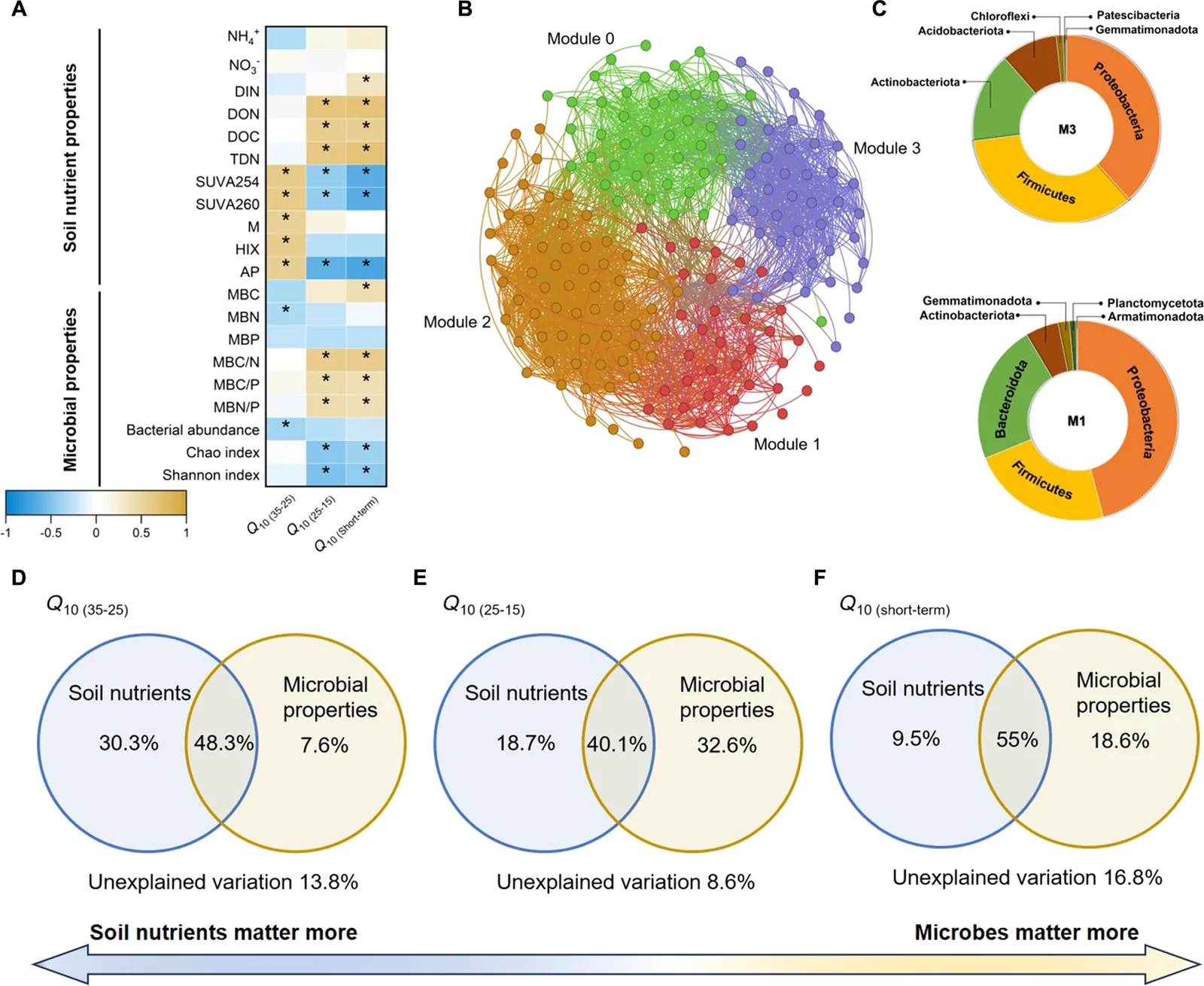
Effects of abiotic and biotic factors on virus-mediated *Q*_10_. (A) Correlation heatmap between explanatory factors (i.e., soil nutrient and microbial properties) and *Q*_10_. (B) Co-occurrence network of bacterial taxa. (C) Bacterial community composition of module 3 and module 1. (D to F) Variation partitioning analysis for *Q*_10_. In (A), *Q*_10 (35-25)_ and *Q*_10 (25-15)_ represent the *Q*_10_ at a higher temperature range (25 to 35 °C) and a lower temperature range (15 to 25 °C) after the 365-d incubation, respectively. *Q*_10 (short-term)_ indicates the *Q*_10_ after the 30-d incubation. In (D) to (F), soil nutrients and microbial properties denote the significantly correlated factors from the correlation heatmap shown in (A) (i.e., DIN, DON, DOC, TDN, SUVA254, SUVA260, M, HIX, AP, MBC/P, MBN/P, MBC/N, bacterial abundance, Chao index, and Shannon index) and the abundance of microbial taxa in 2 microbial ecological modules (M1 and M3). The numbers in the overlapping area of the 2 circles are the combined effects of the 2 factors, and in each circle are the unique effects of the corresponding factor. NO_3_^−^, soil nitrate-N; NH_4_^+^, soil ammonium-N; AP, available phosphorus; DOC, dissolved organic carbon; DON, dissolved organic nitrogen; DIN, dissolved inorganic nitrogen; TDN, total dissolved nitrogen; HIX, humification index of DOM; M, relative molecular mass of DOM; SUVA254, aromatic degree of DOM; SUVA260, hydrophobicity of DOM; MBC, microbial biomass carbon; MBN, microbial biomass nitrogen; MBP, microbial biomass phosphorus; Chao index, richness of bacterial community; Shannon index, α diversity of bacterial community.

Most of the microbial properties were significantly associated with *Q*_10 (short-term)_ and *Q*_10 (25-15)_, whereas only MBN and bacterial abundance showed a correlation with *Q*_10 (35-25)_. Although viruses did not significantly alter the overall microbial community (Fig. S11), 2 distinct microbial ecological clusters (modules) were identified that displayed correlations with *Q*_10_, wherein module 1 (M1) was positively related to *Q*_10 (short-term)_ and *Q*_10 (25-15)_, while module 3 (M3) was positively associated with *Q*_10 (35-25)_ (Fig. 3B and C and Fig. S12). This suggests that soil viruses regulate *Q*_10_ mainly by targeting key microbial taxa in these 2 modules, rather than indiscriminately affecting the overall microbial community. This is consistent with previous studies that found that only some microbial keystone taxa regulate *Q*_10_, although C mineralization is a generalist function performed by almost all microbial taxa [45–48]. It is worth noting that viruses influence *Q*_10_ primarily by first infecting microbes. Consequently, although the net effect of viruses on *Q*_10_ is soil-dependent, the initial alteration of the microbiota is a necessary first step. This mechanism accounts for the universal and primary correlation observed between microbial community metrics and *Q*_10_, irrespective of soil type. Moreover, all recalcitrant DOM components (SUVA254, SUVA260, and HIX) showed significant negative relationships with M1 and positive relationships with M3 (Fig. S13). The remaining 2 microbial ecological modules (0 and 2) were only significantly correlated with HIX (Fig. S13), suggesting that the HIX components can be utilized by most microorganisms (i.e., all 4 microbial ecological modules), whereas SUVA254 and SUVA260 components are only associated with microorganisms that are influenced by soil viruses (i.e., only M1 and M3). Consequently, SUVA254 and SUVA260 were deemed better indicators of virus-induced soil C release and *Q*_10_ values than HIX.

### Drivers of virus-mediated *Q*_10_ variation with temperature

Unraveling the main determinants controlling viral effects on *Q*_10_ is fundamental to understanding the mechanisms underlying virus-triggered soil C-climate feedbacks. Variation partitioning analysis (VPA) for *Q*_10_ was conducted to assess the relative contributions to *Q*_10_ regulation. The 2 groups of factors (i.e., soil nutrient and microbial properties) explained 86.2% of *Q*_10 (35-25)_, 91.4% of *Q*_10 (25-15)_, and 83.2% of *Q*_10 (short-term)_ variances (Fig. 3D to F). The relative importance of nutrient properties and microbial factors as drivers of *Q*_10_ shifted dynamically between nutrient properties and microbial factors during long-term incubation, depending on the temperature conditions. Specifically, soil nutrients played a greater role than microbial properties for *Q*_10 (35-25)_ (soil nutrients = 30.3% versus microbial properties = 7.6%), whereas microbial properties played a greater role for *Q*_10 (25-15)_ (soil nutrients = 18.7% versus microbial properties = 32.6%) (Fig. 3D and E). The short-term incubation also highlighted the importance of microbial properties on the viral mediation of *Q*_10_, with the microbial effect being almost twice that of the soil nutrient effect (Fig. 3F). These results indicate a stronger control of microbial properties by soil viruses governing *Q*_10_ at lower temperatures [*Q*_10 (25-15)_] and shorter incubation times [*Q*_10 (short-term)_], whereas the virus influence on microbial properties was lower at higher temperatures [*Q*_10 (35-25)_].

The change in the dominant regulating factor influencing *Q*_10_ dynamics is ascribed to the response of the viral inherent lifestyle to temperature and soil nutrient availability under long-term incubation. Specifically, soil virus–host interactions and viral infectivity and lysis efficiency are stimulated with increasing temperature below their optimal temperature range (15 to 25 °C in this study). This is further highlighted when considering the effect of viruses on microbial soil C release [49,50]. Once the optimal temperature (35 °C in this study) is exceeded, the viral decay rate is significantly enhanced, and the viral replication rate and infection capacity are significantly weakened, thereby resulting in decreased virus–host interactions [35,51]. The continued depletion of soil C throughout the long-term incubation can also affect *Q*_10_, as the degradation of progressively more recalcitrant C substrates increases *Q*_10_ due to their higher activation energy [31,44,52,53]. Consequently, soil nutrient levels, including C, N, and P, became the main determinant of virus-affected *Q*_10_ at the higher temperature regime (25 to 35 °C) under long-term incubation, whereas virus-affected *Q*_10_ at the lower temperature regime (15 to 25 °C) and short-term incubation is mainly controlled by soil microbial properties.

### Virus-regulated microbial metabolism change with temperature

To elucidate whether shifts in viral infection and lysis efficiency underlie the distinct mechanisms of viral control over *Q*_10_ observed during the 365-d incubation, the abundance of functional genes involved in C, N, and P cycling, as well as other microbial metabolic functions, was assessed using metagenomics (Figs. 4 and 5 and Fig. S14). At 35 °C, the abundance of functional genes related to C mineralization was higher in V groups than in CK groups (SM and DV), whereas at 15 and 25 °C, the abundance of functional genes was lower in the V groups compared to the SM and DV groups for most soil types. The N and P cycle among different treatments also shows the same trend (Note S3 and Fig. S14). In addition, the relative abundance of soil viruses reprogramming microbial metabolic pathways to produce progeny viruses increases significantly at 15 °C, but then gradually decreases as the temperature rises (Fig. S14C). This includes metabolic pathways related to triggering the virus turn into lytic cycle (e.g., propanoate metabolism), synthesizing viral capsid proteins (e.g., lysine biosynthesis), viral DNA replication (e.g., cobalamin transport and metabolism, mismatch repair, DNA replication, and homologous recombination), and the energy required for viral replication (e.g., lipoic acid metabolism) [54–59].

**Fig. 4.**
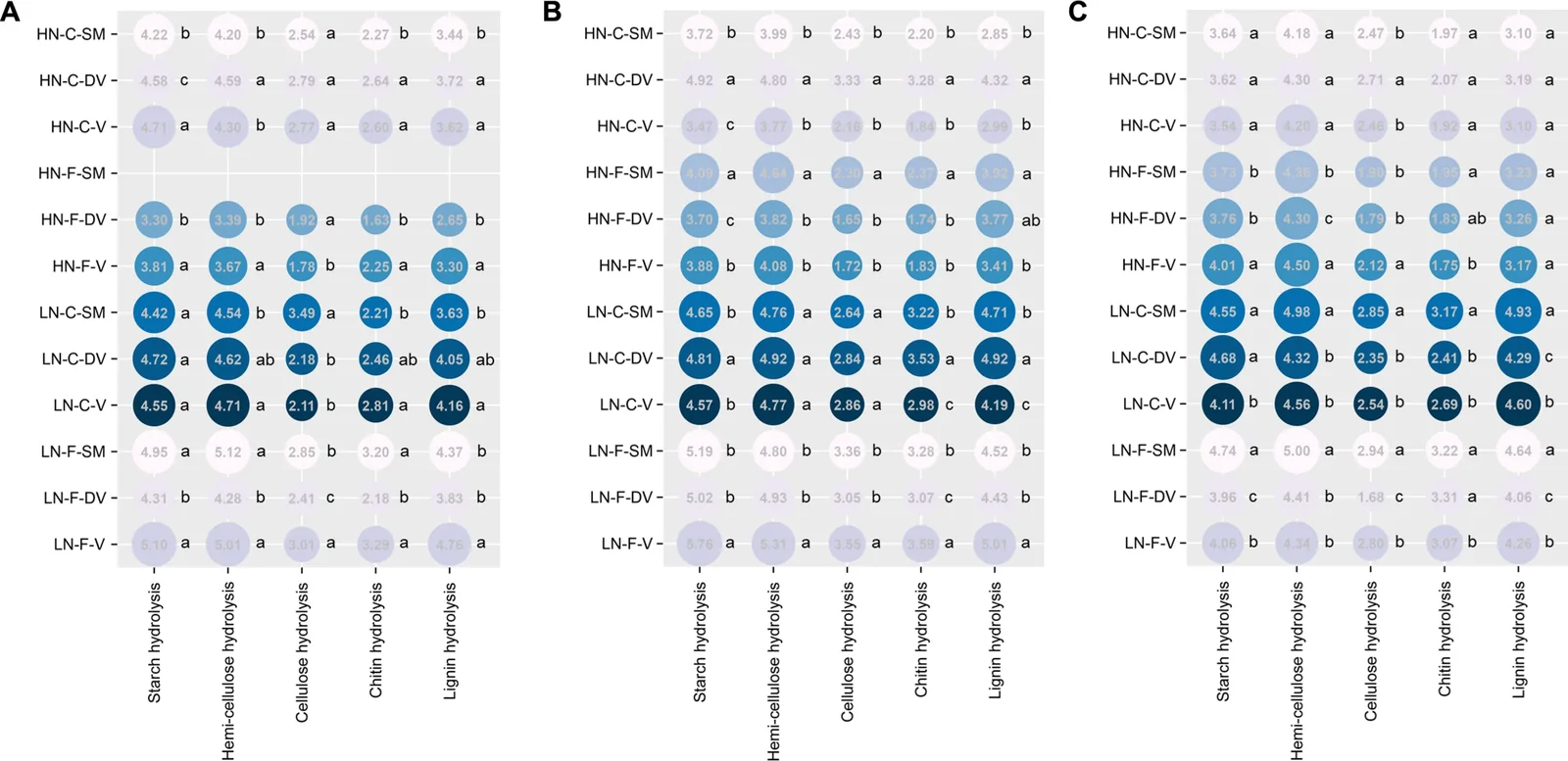
Difference in normalized functional genes related to C degradation with soil viruses at 35 °C (A), 25 °C (B), and 15 °C (C), after long-term incubation. The abundances of the genes were normalized by microbial biomass (i.e., expressed as the copy number μg^−1^ MBC) and then presented as log values. The complexity of C is presented in order from labile to recalcitrant C forms. SM, no added viruses; DV, inactivated viruses; V, active viruses. HN-C/LN-C and HN-F/LN-F represent the incubated soils from cropland and forest sampled in Hunan and Liaoning provinces, China, respectively. Different lowercase letters represent significant differences at *P* < 0.05. The missing data for HN-F-SM at SM in (A) were below the detection limit of the high-throughput quantitative-PCR-based chip (QMEC) analysis.

**Fig. 5.**
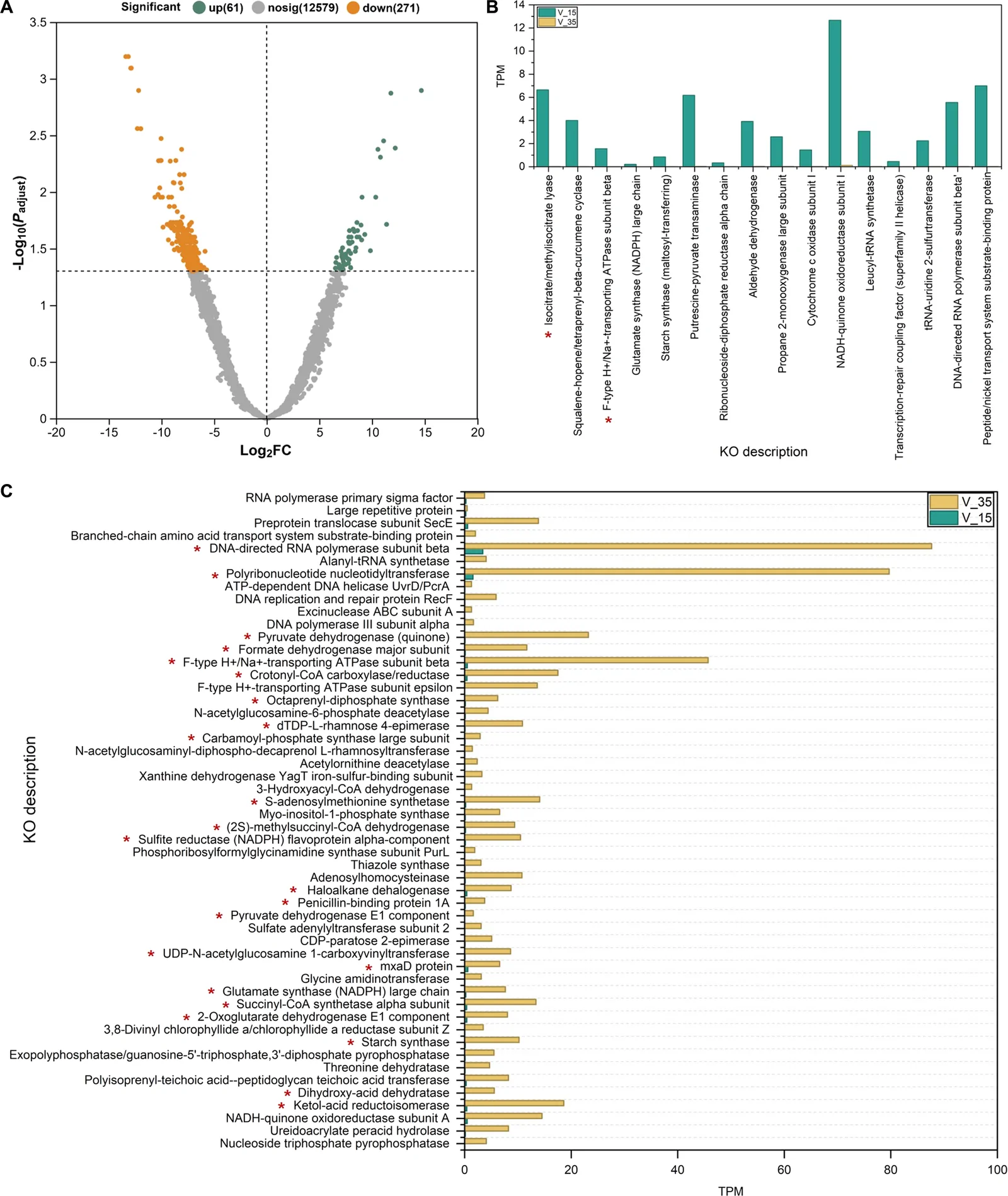
Difference in microbial gene expression levels in the V group at 15 °C compared to the V group at 35 °C, after long-term incubation. (A) The volcano plot shows the differences in gene expression levels between the 2 groups. (B) Functions of genes with significantly higher expression levels in the V group at 15 °C. (C) Functions of genes with significantly lower expression levels in the V group at 15 °C. The red asterisk indicates that this gene’s function also exhibited significant changes in no-virus groups (SM). It was therefore determined that the different temperatures, rather than the virus, affected the microbial metabolism. Consequently, this function comparison was excluded, and only the changes in the microbial metabolic system caused by the virus at 15 and 35 °C were compared.

This implies that higher temperatures (35 °C) reduce viral lysis and replication efficiency, resulting in higher microbial activities with increased abundance of functional genes per unit of microbial biomass in V groups. Thus, *Q*_10_ is mainly influenced by nutrients at higher temperatures. However, the efficiency of viral lysis and replication increased with suppressed microbial activity and lower abundance of functional genes per unit of microbial biomass at lower temperatures (15 °C), resulting in the *Q*_10_ being mainly influenced by microbial abundance and activity.

### RNA insight into viral reprogrammed microbial metabolism

The observed increases in viral lysis and replication efficiency at lower temperatures prompted direct transcriptional profiling via metatranscriptomics to confirm this response at the gene expression level. The results showed that, within the optimal incubation temperature range, viral infectivity and replication increased significantly with rising temperature (Note S4 and Fig. S15). However, once this range is exceeded, viral impact decreased significantly, and microorganisms mainly up-regulated genes related to high-temperature resistance (Fig. S16). This phenomenon was particularly evident when comparing 15 °C with 35 °C. Compared to the V group at 35 °C, the expression levels of 61 genes were significantly up-regulated and those of 271 genes were significantly down-regulated in the V group at 15 °C (Fig. 5A). To accurately determine the effect of the virus on the expression levels of microbial metabolic genes at different temperatures, the influence of temperature on the gene expression of the microorganisms themselves was excluded. Thus, the functions of microbial gene expression levels that changed with temperature in the control group (SM) were not considered, which are marked with asterisks in Fig. 5B and C. At 15 °C, soil viruses stimulate the up-regulation of metabolic functions related to their own replication (Fig. 5B). These functions provide a material basis for the replication of viral proteins and DNA {e.g., glutamate synthase [reduced form of nicotinamide adenine dinucleotide phosphate (NADPH)] large chain and ribonucleoside-diphosphate reductase α chain}, provide energy support [adenosine triphosphate (ATP)] for viral replication [e.g., reduced form of nicotinamide adenine dinucleotide (oxidized form) (NADH)-quinone oxidoreductase subunit I, aldehyde dehydrogenase], and promote viral infection (e.g., tRNA-uridine 2-sulfurtransferase) [60–63].

The metabolic pathways that are significantly down-regulated mainly involve pathways related to microbial response to heat stress (such as nucleoside triphosphate pyrophosphatase, NADH-quinone oxidoreductase subunit A, and glycine amidinotransferase), ensure intracellular energy and substance homeostasis (including adenosylhomocysteinase and thiazole synthase), as well as maintain the integrity of the cell wall structure (e.g., UDP-N-acetylglucosamine-N-acetylmuramyl-pentapeptide-pyrophosphoryl-undecaprenol N-acetylglucosamine transferase and N-acetylglucosamine-6-phosphate deacetylase) (Fig. 5C) [64–69]. All of these findings further demonstrate that the virus has a stronger impact on the metabolic systems of host microorganisms at lower temperatures (15 °C) than at higher temperatures (35 °C). This also confirmed that, from an RNA perspective, the viral infection and lysis efficiency were higher at lower temperatures, which resulted in different underlying mechanisms of *Q*_10_ viral control with temperature changes.

### Limitations and implications

This study substantially advances our understanding of soil virus-mediated C cycling and provides, for the first time, a temperature-resolved perspective on how viruses affect microbial metabolism and *Q*_10_. Our findings offer direct evidence that viral activity modulates *Q*_10_, with the direction of its effect being temperature-dependent—a critical insight for improving predictions of soil C-climate feedbacks under global warming.

Nevertheless, several limitations should be acknowledged. First, current methodological limitations preclude the direct quantification of key viral parameters, such as lysis efficiency and replication activity, which were instead inferred from metagenomic and metatranscriptomic data. Future studies should aim to develop novel approaches for the in situ assessment of these processes. Second, the sample size, though sufficient for controlled incubations, remains limited relative to the diversity of soil types at regional or national scales. Additionally, microcosm-based incubation systems only partially simulate real-world conditions. For instance, soil sterilization by γ-irradiation may alter physicochemical properties, while autoclaving, used here to inactivate viruses, can introduce additional organic matter and nutrients, potentially confounding *Q*_10_ estimates. This is particularly important for *Q*_10_ research. Furthermore, climatic variables, which fluctuate dynamically in natural settings, were also held constant in our experiment, yet they are known to strongly modulate viral–host interactions and C cycling. Soil environment is also a key influencing factor. Therefore, field-based validation will be essential to confirm the ecological relevance of our conclusions. Finally, this work did not account for transitions between different viral life-cycle states (e.g., lysogenic versus lytic phases), which have been shown to differentially influence soil C dynamics and could help explain the contrasting viral effects on *Q*_10_ observed across temperatures [11,12]. Incorporating transformation of viral life-history strategies into future experimental and modeling frameworks will likely yield deeper mechanistic insights.

## Conclusion

Overall, the results of the short- and long-term incubations showed that soil viruses had an important effect on soil C release and *Q*_10_, with maximum changes of nearly 29% and 43%, respectively. Soil viruses were found to significantly decrease *Q*_10_ under short-term incubation, regardless of soil type; however, no consistent patterns were observed under long-term incubation. Besides, the direction of viral impact on *Q*_10_ was completely reversed for all soils across the 2 temperature ranges (15 to 25 °C versus 25 to 35 °C). This reversal depended on shifts in the relative influence of viruses on microbial and nutrient properties at temperatures below and above their optimal growth range. At lower temperatures, viruses exhibited enhanced efficiency in host cell infection and lysis, leading to a more pronounced reprogramming of microbial metabolism to support viral replication. This was accompanied by a significant up-regulation in the synthesis of viral capsids, DNA replication, and overall infection capacity. Conversely, elevated temperatures diminished viral impacts, as evidenced by a marked reduction in the abundance of genes related to viral protein synthesis and DNA replication. Thus, temperature-dependent viral regulation of microbial metabolism led to shifts in the key drivers of *Q*_10_, ranging from microbial properties to soil nutrients. The contrasting temperature-driven effects of viruses on soil C emissions greatly advanced our understanding of how viruses influence global C cycling under rapid global change.

## Materials and Methods

### Soil sampling and extraction of soil viral and bacterial suspensions from soils

To reveal a more universal rule, we collected soils from natural forests (HN-F, LN-F, HAN-F, HEN-F) and croplands (HN-C, LN-C, HAN-C, HEN-C) across 4 Chinese provinces (i.e., Hunan, Liaoning, Hainan, and Henan), where soil type, natural environment [i.e., mean annual precipitation (MAP) and mean annual temperature (MAT)], microbial community, and human disturbance all differed substantially. The soils in Hunan and Hainan provinces belong to the red soil series, while the soils in Henan and Liaoning provinces are brown soil. The MAT and MAP of the sampling sites were retrieved from the WorldClim database (https://www.worldclim.org/), which are listed in Table S3. During soil sampling, five 2 m × 2 m square subplots were established at each corner and in the middle of 3 randomly selected sites for each soil type. The 15 subplot samples (0 to 20 cm) were combined to form a single, equally weighted composite sample representative of the study area, and then transported to the laboratory on ice. After passing through a 2-mm sieve, the soil was divided into 2 parts: one air-dried for sterilization by 50 kGy γ-radiation for 24 h, and the other stored at 4 °C for extraction of soil viruses and bacteria.

Soil viruses and bacterial suspensions were extracted according to a previous study [22]. Specifically, 2 kg of soil was suspended in 2 l of saline magnesium buffer (SM). After vortexing, shaking, and centrifugation, the suspension was filtered and concentrated by tangential flow filtration (TFF) using 0.22-μm and 100-kDa filters. Retentates (>0.22 μm) were collected and treated as bacterial suspensions, and the concentrated filtrates were used as viral suspensions (100 kDa–0.22 μm) and virus-free suspensions (<100 kDa). The concentrations of viral and bacterial suspensions were calculated and then added to each serum vial containing 20 g of sterilized air-dried soil of the different soil types [19]. Soil moisture was maintained at 40% of field capacity throughout the incubation by the addition of sterilized water (filtered through 100 kDa).

### Incubation experimental design

Incubations were conducted in 250-ml serum bottles with 20 g of sterilized air-dried soil (50 kGy γ-radiation for 24 h), with treatments consisting of (a) bacterial suspensions with no added phage (SM), (b) bacterial suspensions with autoclaved dead phage (DV), and (c) bacterial suspensions with active virus (V). Prior to the experiment, all microcosms underwent a 30-d pre-incubation at 25 °C to reactivate the microbial and viral communities and allow for acclimation to the experimental conditions. Subsequently, they were subjected to short-term (30 d) and long-term (365 d) incubations at 3 temperatures (15, 25, and 35 °C) to quantify the effects of viral activity on soil C release and *Q*_10_. The 3 temperatures were selected to (a) facilitate a comparison of viral effects on soil C release and *Q*_10_ with corresponding results from previous studies [22,28,29], and to verify whether the results of viruses decreasing *Q*_10_ were similar to other soil types when incubated within the optimal viral and microbial growth temperature range (15 to 25 °C), and (b) investigate how the effects of soil viruses on soil C release and *Q*_10_ change when the incubation temperature is increased beyond the optimal temperature (up to 35 °C) [9,32,35,70]. The combination of short- and long-term incubations allowed us to capture the viral effects on the temperature response of soil C release, as well as the long-term virus-regulated C dynamics in response to prolonged warming. The short-term incubation experiment comprised a total of 216 microcosms. This number was calculated from 3 factors: 3 viral treatments (SM, DV, and V) × 3 incubation temperatures (15, 25, and 35 °C) × 8 soil types × 3 replicates. Therefore, considering the substantial workload and the need for representative conclusions, we integrated all 3 criteria (i.e., soil type, climatic conditions, and soil nutrient content) and ultimately selected soils from Hunan and Liaoning provinces for long-term incubation. Consequently, the long-term experiment involved 108 microcosms (3 treatments × 3 temperatures × 4 soils × 3 replicates). Other detailed methods are given in the Supplementary Materials.

## Data Availability

All data needed to evaluate the conclusions of this paper are present in the paper and the Supplementary Materials. The original sequence data were deposited into the National Center for Biotechnology Information Sequence Read Archive database under project ID PRJNA1534627 and PRJNA1546981. Additional data related to this paper may be requested from the authors.

## Supplementary Materials

Supplementary 1 Materials and Methods Notes S1 to S4 Figs. S1 to S16 Tables S1 to S3
