# Supplementary material for: The Temperature Sensitivity of Soil CO2 Release Is Modified by Virus–Bacteria Interactions
Source: Research (Wash D C). 2026 Nov 13;9:1479. doi: 10.34133/research.1479 (PMC13760055; doi:10.34133/research.1479)
Supplement: Supplementary file 1 [file research.1479.f1.docx]

**Supplementary Materials for**

## The temperature sensitivity of soil CO_2_ release is modified by virus-bacteria interactions

Jianming Xu ^1,2*^, Di Tong ^1,2^, Jiachun Shi ^1,2^, Junjie Xie ^1,2^, Nan Zhang ^1,2^, Zhongmin Dai ^1,2,3^, Shuai Zhang ^1^, Randy A. Dahlgren ^4^, Bin Ma ^1,2^

^1^State Key Laboratory of Soil Pollution Control and Safety, College of Environmental and Resource Sciences, Zhejiang University, Hangzhou, Zhejiang, China

^2^Zhejiang Provincial Key Laboratory of Agricultural Resources and Environment, Zhejiang University, Hangzhou, Zhejiang, China

^3^The Rural Development Academy, Zhejiang University, Hangzhou, Zhejiang, China

^4^Department of Land, Air and Water Resources, University of California, Davis, CA, USA

*Corresponding author: Jianming Xu

**E-mail:** [jmxu@zju.edu.cn](mailto:jmxu@zju.edu.cn)

**This PDF file includes:**

Materials and Methods

Supplementary Notes 1 and 4

Figures S1 to S16

Table S1 to S3

References

## Materials and Methods

## Calculation of Q_10_ under long-term incubation

All samples were preincubated at 25℃ for 30 days to allow for microbial and viral resuscitation and to exclude pulses of microbial activity due to the release of nutrients following soil sterilization and moisture adjustment [1]. During the following 365-day incubation, the CO_2_ emission rate was determined by gas chromatography (GC-200 Plus Shimadzu, Japan) every 2 to 4 days in the first month, then weekly in the second month, and monthly thereafter. The soil C release rate (mg CO_2_ kg soil^-1^ d^-1^) and cumulative C release were calculated as in a previous study [8]. After normalizing the cumulative C release from the long-term incubation to a per unit OC basis, the long-term Q_10_ for two temperature ranges (35 versus 25℃ and 25 versus 15℃) was calculated as follows [9, 10]:

$C_{cum}\left( t \right)= C_{0} \times(1-e^{\left( -kt \right)}$)	(1)

$Q_{10}= \left( \frac{k(T_{w})}{k(T_{c})} \right)^{\frac{10}{T_{w}-T_{c}}}$	(2)

where C_cum_(t), C_0_, and k represent the cumulative amount of C released at time t (mg CO_2_-C g^−1^ OC), preincubation SOC content (mg C g^−1^ OC), and the decay rate constant (day^−1^), respectively. The model fitted the incubation data well (*R^2^* > 0.93, *P* < 0.0001; Table S1). Soil Q_10_ at the higher (25-35°C) and lower (15-25°C) temperature ranges was then calculated as the ratio of the decay rate constant, respectively.

**Calculation of Q_10_ for short-term incubation**

Soil samples were also incubated at 15, 25, and 35°C for 30 days using identical conditions to the 365-day incubation. The rate of CO_2_ release at each temperature was determined as in the long-term incubation and then fitted to Q_10_ using a first-order exponential equation [11].

$R=Be^{kT}$	(3)

$Q_{10}= e^{(10k)}$	(4)

where R is the soil CO_2_ release rate at a given temperature (mg CO_2_-C g^-1^ OC d^-1^), T is the incubation temperature (°C), and B and k are fitted parameters. Based on equation (3), Q_10_ for the short-term incubation (Q_10 (short-term)_) was calculated from equation (4). The equation described the relationships well for all soils (*R^2^* > 0.65; Table S2).

## Soil property characterization

Dissolved organic carbon (DOC) and total nitrogen (TDN) were extracted with MilliQ water at a ratio of 1:10 (w/v), filtered through a 0.45 μm membrane filter, and measured using a Multi C/N TOC analyzer. Soil NH_4_^+^ and NO_3_^-^ were extracted from fresh soil with 1 M KCl and measured using a flow injection analyzer [12]. Dissolved inorganic N (DIN) was calculated as the sum of NH_4_^+^-N and NO_3_^-^-N, and dissolved organic N (DON) was calculated as the difference between total dissolved nitrogen (TDN) and DIN (i.e., DON = TDN - DIN) [13]. Available P (BrayP) was quantified using the molybdenum blue spectroscopy method after extraction with 0.03 M NH_4_F/0.025 M HCl [14]. Microbial biomass carbon (MBC), nitrogen (MBN), and phosphorus (MBP) were determined using CHCl_3_ fumigation-extraction [15, 16].

The aromaticity (SUVA254), hydrophobicity (SUVA260), and relative molecular weight (M) of DOM were determined using an ultraviolet-visible spectrophotometer [17, 18]. SUV254 and SUV260 were calculated as the ratio of UV absorbance at 254 and 260 nm to the DOC concentration [19]. After dilution of DOC concentrations to ~8 mg C L^-1^, excitation-emission matrix (EEM) spectra of DOM were measured by fluorescence spectrometry at a scan rate of 2000 nm min^-1^ and further analyzed and visualized in MATLAB 2012b using the DOM Fluor toolbox [19]. The humiﬁcation index (HIX) was calculated for an excitation wavelength of 254 nm by dividing the peak area of the emission wavelengths between 435 and 480 nm by the sum of peak areas between 435-480 nm and 300-345 nm [20].

## DNA extraction, quantitative PCR, and 16S rRNA amplicon sequencing

Soil DNA was extracted using the MoBio PowerSoil DNA Isolation Kit (MP Biomedicals, USA) according to the manufacturer's instructions. The absolute abundance of bacteria was determined by quantitative polymerase chain reaction (qPCR). The PCR mixture contained 1 μL of DNA template, 10 μL of TB Premix Ex Taq (Takara, Japan), 0.8 μL of primer F/R (10 μM), and 7.4 μL of double-distilled H_2_O. The primers 515F (GTGCCAGCMGCCGCGGTAA) / 806R (GGACTACHVGGGTWTCTAAT) were used for qPCR with reaction conditions as follows: 94 °C for 3 min followed by 40 cycles of 94 °C for 15 s, 56 °C for 35 s, and 72 °C for 1 min [21]. Gene copy numbers were determined from standard curves generated from six concentration levels of plasmid DNA. Negative controls were prepared for each assay using sterilized H_2_O [22].

The V3-V4 region of the 16S rRNA gene was amplified by primers 338F (ACTCCTACGGGAGGCAGCAG) / 806R (GGACTACHVGGGTWTCTAAT) and sequenced on a HiSeq PE250 platform (Illumina, USA) [23, 24]. Quantitative Insights into Microbial Ecology 2 (QIIME2) was used to process the raw sequences [25]. After removing barcodes and primers, the raw reads from each sample were merged into paired-end reads, and then trimmed, denoised, and clustered into amplicon sequence variants (ASVs) using the DADA2 pipeline in QIIME2 [26]. Taxonomic annotation for each bacterial ASV was performed using SILVA (version 138) [27].

## Determination of functional gene abundances

High-throughput quantitative-PCR-based chip (QMEC) was applied to quantify the absolute abundance of functional genes related to C, N, and P mineralization [28]. Abundances of functional genes involved in the degradation of starch (*amyA*, *apu*, *sga*, and *iso-plu*), hemicellulose (*xylA*, *abfA*, and *manB*), cellulose (*cex*), chitin (*chiA*), lignin (*lig*, *glx*, and *mnp*), organic P (*bpp*, *phnk*, and *phoD*), and organic N (*gdhA*) were normalized by microbial biomass carbon and expressed as the copy number μg^−1^ MBC, to remove the influence of microbial biomass content on gene abundance [11]. Three technical replicates of each primer set were performed along with a non-template negative control in each run.

## Metagenomic processing protocols

Sequencing libraries were generated using the Illumina TruSeq Nano DNA LT Library Preparation Kit and then sequenced with an Illumina NovaSeq 6000 (Illumina, USA), according to the manufacturer’s recommendations. Trimmomatic (v2.39) and BWA-MEM (v0.7.17) were used to process the raw reads and remove the eukaryotic genome sequence, respectively, during which the short (< 50 bp) and low-quality reads (< 20 bases) were filtered[29, 30]. Then, the metagenomic reads from each sample were de novo assembled using MEGAHIT68 545 (v.1.2.9) with a minimum contig length of 500 bp (k-step = 10; k-min = 27)^[31]^. The open reading frames (ORFs) were predicted by using Prodigal (v2.6.3) with default parameters, and the redundant scaffolds were removed using the Linclust program. Classification of prokaryotic sequences was performed by CLARK with the Genome Taxonomy Database (GTDB)[32, 33]. To calculate the abundance of contigs or genes, CoverM was used to calculate the number of reads aligned per contig/gene base (--methods reads_per_base) with strict parameters (--min-read-percent-identity 0.99 --min-read-aligned-percent 0.75) for accuracy[34, 35]. The read number for each sample was determined using seqkit2 (algorithm: stat) for normalizing the relative abundance by read per kilobase per million mapped reads (RPKM). The functional genes and pathways were annotated using the Kyoto Encyclopedia of Genes and Genomes (KEGG) database (http://www.genome.jp/kegg/) with an e-value cut-off of 1e-5.

## RNA extraction and Metatranscriptomic processing protocols

Total RNA was isolated and purified using a Soil RNA Extraction Kit (Majorbio, Shanghai, China), following the manufacturer's procedure. The quantity and integrity of the extracted RNA were measured using a NanoDrop 2000 spectrophotometer (Thermo Scientific, Massachusetts, USA) and an Agilent 5300 Bioanalyzer (Agilent Technologies, California, USA). The RNA underwent standard Illumina library preparation using an Illumina® Stranded mRNA Prep Ligation Kit (Illumina, San Diego, CA, USA) and rRNA was depleted using a RiboCop™ rRNA Depletion Kit for Mixed Bacterial Samples (Lexogen, USA). Sequencing was performed using the paired-end method on an Illumina Novaseq X Plus sequencer (Illumina, San Diego, CA, USA) at Majorbio Bio-Pharm Technology Co., Ltd. (Shanghai, China).

Quality control of the raw data generated by sequencing was carried out using fastp (https://github.com/OpenGene/fastp, v. 0.23.4), which included reads of less than 50 base pairs and ends with a Phred quality score of less than 20 [36]. SortMeRNA (http://bioinfo.lifl.fr/RNA/sortmerna/, v. 4.3.6) was used to eliminate rRNA and MEGAHIT (v. 1.2.9) was used for de novo assembly and splicing [37, 38]. All transcripts assembled from each sample with a length ≥500 bp were merged using CD-HIT (http://www.bioinformatics.org/cd-hit/, v.4.6.1) with an identity of 0.95 and minimum coverage of 0.9 to remove redundancy [39], and the longest sequence was used as the representative sequence of the unigene. Unigene abundance for a given sample was estimated using TPM, based on the number of aligned reads obtained with RSEM (http://deweylab.biostat.wisc.edu/rsem/, version 1.3.3). The best-hit taxonomy of the unigenes was obtained by aligning them against the NCBI NR database using DIAMOND (http://www.diamondsearch.org/index.php; version 2.0.13) with an e-value cut-off of 1e-5. Similarly, the KEGG functional annotation of unigenes was obtained. Based on the taxonomic and functional annotation of the unigenes, as well as their abundance profile, differential analysis was carried out at each taxonomic, functional or gene-wise level using a Kruskal–Wallis test.

## Statistical analyses

The response ratio of the measured properties to soil viruses was calculated using the following equation:

$Response \left( \% \right)=\left( P_{V}- P_{CK} \right)/{P_{CK}}\times100$	(5)

where *P_V_* is the measured property in the virus group (V) and *P_CK_* is the property in the control groups (SM or DV).

*Vegan* package was applied to determine bacterial alpha diversity (Chao index and Shannon index). The bacterial community composition was assessed by principal coordinates analysis (PCoA) based on a Bray-Curtis dissimilarity matrix [40]. Bacterial taxa at the genus level with a relative abundance greater than 0.01% were selected, and then pairwise Spearman's rank correlations between all bacterial taxa were calculated using *psych* package [41]. The bacterial co-occurrence network was considered stable only when Spearman's correlation was > 0.5 and p < 0.01. Ecological clusters (modules) of soil bacterial taxa were identified using the default parameters of Gephi. The relative abundance of each module was calculated by averaging the standardized relative abundances (Z-score) of all ASVs belonging to each module [42].

Correlation heatmaps and other plots were visualized using Origin. One-way ANOVAs followed by least-signiﬁcant difference (LSD) tests were used to determine differences between measured parameters (e.g., cumulative CO_2_ release, Q_10_, SUV254, HIX, and SUV260). SPSS version 24 (SPSS, Chicago, IL, USA) and R version 3.6.3 were used for statistical analyses with a significance level of *p* < 0.05 unless otherwise stated.

# Supplementary Notes 1. Impact of soil viruses on C release

In contrast to previous studies that focused primarily on viral effects on CO_2_ emissions over short incubation periods [2, 3], we conducted a long-term incubation to investigate how and to what extent soil viruses influence soil C release and Q_10_ dynamics. In general, whether the incubation temperature was within (i.e., 15 and 25°C) or above (i.e., 35°C) the optimum temperature range for viral and microbial growth [6, 7], the cumulative C release was notably enhanced with increasing temperature (Supplementary Fig. S1), regardless of soil type or treatment. Soil virus impacts on C release ranged from -29% to 18%; Supplementary Fig. S2 D-F), indicating that the viral effect on soil C release was not consistent across soils, and that the cumulative CO_2_ emission in the active virus groups was likely to be either higher and lower than in the control (CK) groups (SM and DV) (Supplementary Fig. S2). This result agrees with previous studies that found a large difference in viral impacts on soil C release across different soil types [2, 43].

# Supplementary Notes 2. Key drivers of C release mediated by soil viruses

After excluding the influence of mineral protection, we assessed the role of soil nutrients and microbial properties in regulating C release. Viral effects on soil N cycling were significantly correlated with C release, with soil NH_4_^+^-N and NO_3_^-^-N showing a positive correlation and DON displaying a negative correlation (Supplementary Figs. S7-S9). This implies that DON in the lysate materials was mineralized, thereby becoming available for utilization by the surviving microbes or accumulating as inorganic nitrogen [44, 45]. Similarly, soil viruses contributed to changes in soil P cycling [1, 46]. We found negative correlations of cumulative C release with microbial biomass phosphorus (MBP) and available phosphorus (AP), and a positive correlation with microbial biomass stoichiometric ratios (i.e., MBC/N, MBC/P and MBN/P). This suggests a divergence of microbial C/N/P stoichiometry caused by viral lysing that results in a feedback on soil C release [46]. Previous studies using a biophysical scaling model suggested that viral lysis can result in P limitation for microorganisms, as P originating from lyses of microbial hosts is preferentially allocated to virions relative to C and N (e.g., 33% P vs. only 10% of C content in bacteria) [47].

To assess whether the recycling of lysate DOM could be associated with the accumulation of NH_4_^+^ and the depletion of DON, AP and MBP, a correlation analysis was conducted (Supplementary Fig. S10). The significant positive association between the indicators of lysate DOM (SUVA254 and SUVA260) with NH_4_^+^, and the negative relationships with other properties (DON, AP and MBP), support the reutilization of lysate DOM. These relationships substantiate the key factors/mechanisms contributing to the changes in soil nutrients and MBP, and ultimately the release of soil C. Available-P limitation could stimulate soil C release as microbes secrete enzymes to acquire additional P [48-50]. This is supported by the negative relationship between AP and MBP with cumulative CO_2_ release, whereas the nutrient properties displayed positive correlations. Collectively, these results highlight the pivotal role of soil viruses in decoupling soil C/N/P cycles and microbial C/N/P stoichiometry.

# Supplementary Notes 3. The N and P cycle among different treatments

The impact of soil viruses on the abundance of functional genes involved in N and P cycling was the total opposite at 35°C and 15°C in the vast majority of soils (Fig. S14 A and B). Lower temperatures tend to have a more inhibitory effect, while higher temperatures tend to have a more stimulating effect.

# Supplementary Notes 4. RNA insight into viral reprogrammed microbial metabolism among 15-25°C and 25-35°C

Compared to the V group at 25°C, 1 gene was significantly upregulated at 15°C and could not be annotated by the KEGG database (Fig. S15). Additionally, the 24 genes that were significantly downregulated at 15°C were associated with viral receptor proteins (e.g., tight adherence protein C) and pathways related to the utilisation of host resources (e.g., small subunit ribosomal protein S2) [51]. The expression of functional genes related to the redirection of the host's resources for viral replication (e.g., DNA-directed RNA polymerase subunit beta, crotonyl-CoA carboxylase/reductase) and the assembly of viral head proteins (e.g., GTP-binding protein and elongation factor G) was upregulated at 25°C, compared to 35°C [52-54]. In addition, the down-regulated genes were mainly involved in pathways related to the microbial response to heat stress (e.g., citrate synthase) [55].

**Fig. S1.** Dynamics of cumulative CO_2_ emission (mean ± std dev) with added viruses incubated over 365 days at three different temperatures. SM, no added viruses; DV, inactive viruses; V, active viruses; 35, 25, and 15 signify different temperatures (35 °C, 25 °C, and 15 °C) during the incubation. HN-C/LN-C (A & C) and HN-F/LN-F (B & D) represent the incubated soils from cropland and forest sampled in Hunan and Liaoning Provinces, China, respectively.

**Fig. S2.** Cumulative CO_2_ release (mean ± std dev) at the end of 365-day incubation period at 35 °C (A), 25 °C (B), and 15 °C (C), and the response ratio of viral effects on cumulative CO_2_ release of control groups (SM, and DV) and V group at 35 °C (D), 25 °C (E) and 15 °C (F). SM, no added viruses; DV, inactive viruses; V, active viruses; HN-C/LN-C and HN-F/LN-F represent soils from cropland and forest in Hunan and Liaoning Provinces, China, respectively. Different lowercase letters represent significant differences at *p* < 0.05.

**Fig. S3**. Response ratio of viral effects on Q_10_ of control groups (SM, and DV) versus V group based on the 365-day incubation (A; higher temperature range (25-35°C) and B; lower temperature range (15-25°C)) and 30-day incubation period (C, and D). SM, no added viruses; DV, inactive viruses; V, active viruses; HN-C/LN-C/HaN-C/HeN-C and HN-F/LN-F/HaN-F/HeN-F represent soils from cropland and forest in Hunan, Liaoning, Hainan, and Henan Provinces, China, respectively.

**Fig. S4**. Response ratio of viral effects on DOM components of control groups (SM and DV) and V group at 35 °C (A), 25 °C (B), and 15 °C (C), after the 365-day incubation. SM, no added viruses; DV, inactive viruses; V, active viruses. HN-C/LN-C and HN-F/LN-F represent soils from cropland and forest in Hunan and Liaoning Provinces, China, respectively. HIX, SUVA254, and SUVA260 indicate the degree of humidification, aromaticity, and hydrophobicity of DOM, respectively. Asterisk indicates a significant difference (*p* < 0.05) between the virus group and control groups (SM and DV). Yellow and blue bars denote positive and negative values, respectively. Length of the bar represents the absolute value of the response ratio.

**Fig. S5**. Differences (mean ± std dev) in iron-bound organic carbon (FeOC) between virus group (V) and control groups (SM and DV) at 35 °C (A), 25 °C (B), and 15 °C (C), after the 365-day incubation. SM, no added viruses; DV, inactive viruses; V, active viruses. HN-C/LN-C and HN-F/LN-F represent soils from cropland and forest in Hunan and Liaoning Provinces, China, respectively.

**Fig. S6**. Relationship between iron-bound organic carbon (FeOC) with cumulative CO_2_ emissions and Q_10_ at 35 °C (A and D), 25 °C (B and E), and 15 °C (C and F) after 365-day incubation. SM, no added viruses; DV, inactive viruses; V, active viruses. HN-C/LN-C and HN-F/LN-F represent soils from cropland and forest in Hunan and Liaoning Provinces, China, respectively. Each point on each graph represents a microcosm sample. Therefore, each graph contains 36 data points in total: 3 soil viral treatments (SM, DV and V) × 4 different soils × 3 replicates.

**Fig. S7**. Relationship of cumulative CO_2_ release with soil nutrient properties (shown in blue) and microbial properties (shown in yellow) at 35 °C after 365-day incubation. NO_3_^-^, nitrate-N; NH_4_^+^, ammonium-N; AP, available phosphorus; DOC, dissolved organic carbon; DON, dissolved organic nitrogen; DIN, dissolved inorganic nitrogen; TDN, total dissolved nitrogen; HIX, humiﬁcation index of DOM; MD, relative molecular mass of DOM; SUVA254, aromatic degree of DOM; SUVA260, hydrophobicity of DOM; MBC, microbial biomass carbon; MBN, microbial biomass nitrogen; MBP, microbial biomass phosphorus; Chao index, richness of bacterial community; Shannon index, alpha diversity of bacterial community. Linear regression lines with 95% confidence intervals; **p* < 0.05, ***p* < 0.01 and ****p* < 0.001. Each point on each graph represents a microcosm sample. Therefore, each graph contains 36 data points in total: 3 soil viral treatments (SM, DV and V) × 4 different soils × 3 replicates.

**Fig. S8**. Relationship of cumulative CO_2_ release with soil nutrient properties (shown in blue) and microbial properties (shown in yellow) at 25 °C after 365-day incubation. NO_3_^-^, nitrate-N; NH_4_^+^, ammonium-N; AP, available phosphorus; DOC, dissolved organic carbon; DON, dissolved organic nitrogen; DIN, dissolved inorganic nitrogen; TDN, total dissolved nitrogen; HIX, humiﬁcation index of DOM; MD, relative molecular mass of DOM; SUVA254, aromatic degree of DOM; SUVA260, hydrophobicity of DOM; MBC, microbial biomass carbon; MBN, microbial biomass nitrogen; MBP, microbial biomass phosphorus; Chao index, richness of bacterial community; Shannon index, alpha diversity of bacterial community. Linear regression lines with 95% confidence intervals; **p* < 0.05, ***p* < 0.01 and ****p* < 0.001. Each point on each graph represents a microcosm sample. Therefore, each graph contains 36 data points in total: 3 soil viral treatments (SM, DV and V) × 4 different soils × 3 replicates.

**Fig. S9**. Relationship of cumulative CO_2_ release with soil nutrient properties (shown in blue) and microbial properties (shown in yellow) at 15 °C after 365-day incubation. NO_3_^-^, nitrate-N; NH_4_^+^, ammonium-N; AP, available phosphorus; DOC, dissolved organic carbon; DON, dissolved organic nitrogen; DIN, dissolved inorganic nitrogen; TDN, total dissolved nitrogen; HIX, humiﬁcation index of DOM; M, relative molecular mass of DOM; SUVA254, aromatic degree of DOM; SUVA260, hydrophobicity of DOM; MBC, microbial biomass carbon; MBN, microbial biomass nitrogen; MBP, microbial biomass phosphorus; Chao index, richness of bacterial community; Shannon index, alpha diversity of bacterial community. Linear regression lines with 95% confidence intervals; **p* < 0.05, ***p* < 0.01 and ****p* < 0.001. Each point on each graph represents a microcosm sample. Therefore, each graph contains 36 data points in total: 3 soil viral treatments (SM, DV and V) × 4 different soils × 3 replicates.

**Fig. S10**. Correlation heatmap between indicators of lysate DOM (SUVA254 and SUVA260) and soil nutrient properties after 365-day incubation. SUVA254, aromatic degree of DOM; SUVA260, hydrophobicity of DOM; MBP, microbial biomass phosphorus; NO_3_^-^, soil nitrate-N; NH_4_^+^, soil ammonium-N; AP, available phosphorus; DON, dissolved organic nitrogen. **p* < 0.05.

**Fig. S11**. Principal coordinates analysis (PCoA) of bacterial communities in different treatment groups after preincubation. SM, no added viruses; DV, inactive viruses; V, active viruses. HN-C/LN-C and HN-F/LN-F represent soils from cropland and forest in Hunan and Liaoning Provinces, China, respectively.

**Fig. S12**. Correlation heatmap between microbial ecological models and Q_10_ based on Spearman correlation. Q_10 (35-25)_ and Q_10 (25-15)_ represent the Q_10_ at the higher (25 – 35 °C) and a lower (15 – 25 °C) temperature ranges after the 365-day incubation period, respectively. Q_10 (short-term)_ indicates the Q_10_ after the 30-day incubation. Ecological clusters (modules) of soil bacterial taxa were identified using the default parameters of Gephi. M0, microbial ecological model 0; M1, microbial ecological model 1; M2, microbial ecological model 2; M3, microbial ecological model 3. **p* < 0.05, ***p* < 0.01 and ****p* < 0.001.

**Fig. S13**. Relationship of microbial ecological models (M0, M1, M2, M3) and DOM components. HIX, SUVA254 and SUVA260 indicate the degree of humidification, aromaticity and hydrophobicity of DOM, respectively. Ecological clusters (modules) of soil bacterial taxa were identified using the default parameters of Gephi. M0, microbial ecological model 0; M1, microbial ecological model 1; M2, microbial ecological model 2; M3, microbial ecological model 3. Linear regression lines with 95% confidence intervals; **p* < 0.05, ***p* < 0.01 and ****p* < 0.001.

**Fig. S14.** Difference in functional genes related to N and P cycling, and other microbial metabolic functions (mean ± std dev) with soil viruses. In panels A and B, gene abundances were normalized by microbial biomass (i.e., expressed as copy number μg^-1^ MBC), and the difference in change between the V and SM groups was then calculated. Red indicates that the addition of the virus enhanced this function, while blue indicates that it inhibited it. Grey indicates that some samples were below the QMEC analysis detection limit and could not be included in the calculation. In panel C, microbial metabolic functions were compared with active viruses added at different temperatures. HN-C/LN-C and HN-F/LN-F represent soils that were incubated from cropland and forest samples taken in Hunan and Liaoning provinces in China, respectively.

**Fig. S15.** Difference in microbial gene expression between the V group at 15°C and the V group at 25°C after long-term incubation. The left volcano plot shows differentially expressed genes between the two groups. The right bar plot on the right shows the functional categories of genes that were significantly altered between 15°C and 25°C in the V group. The red asterisks mark functions that also changed significantly in the no-virus groups (SM), indicating that temperature, rather than viral activity, was the main driver of these changes. Such functions were therefore excluded from the final comparison, allowing specific examination of virus-induced shifts in microbial metabolic systems at 15°C and 25°C. N.S. indicates genes that could not be annotated in the KEGG database.

**Fig. S16.** Difference in microbial gene expression between 25°C and 35°C in the V group after long-term incubation. The left volcano plot shows differentially expressed genes between the two groups. The right bar plot shows the functional categories of genes significantly altered between 25°C and 35°C in the V group. The red asterisks mark functions that also changed significantly in the no-virus groups (SM), indicating that temperature, rather than viral activity, was the main driver of these changes. Such functions were therefore excluded from the final comparison, allowing specific examination of virus-induced shifts in microbial metabolic systems at 25°C and 35°C.

# Supplementary tables

**Table S1.** Parameters obtained for the ﬁtted ﬁrst-order kinetic one-compartment model (C_cum_(t) = C_0_ × (1 – e^-kt^)) for the 365-day incubation data at the three incubation temperatures. HN-C/LN-C and HN-F/LN-F represent soils from cropland and forest in Hunan and Liaoning Provinces, China, respectively. All model *p*-values were <0.0001.

| **Groups** | **35℃** | | **25℃** | | | **15℃** | |
| --- | --- | --- | --- | --- | --- | --- | --- |
|  | ***k*** | ***R^2^*** | | ***k*** | ***R^2^*** | ***k*** | ***R^2^*** |
| HN-C-SM1 | 3.110E-03 | 0.973 | | 2.350E-03 | 0.965 | 1.310E-03 | 0.942 |
| HN-C-SM2 | 3.080E-03 | 0.974 | | 2.260E-03 | 0.965 | 1.320E-03 | 0.938 |
| HN-C-SM3 | 3.110E-03 | 0.974 | | 2.350E-03 | 0.966 | 1.340E-03 | 0.940 |
| HN-C-DV1 | 2.940E-03 | 0.964 | | 2.120E-03 | 0.974 | 1.190E-03 | 0.945 |
| HN-C-DV2 | 2.930E-03 | 0.965 | | 2.140E-03 | 0.972 | 1.180E-03 | 0.946 |
| HN-C-DV3 | 2.970E-03 | 0.967 | | 2.160E-03 | 0.971 | 1.190E-03 | 0.945 |
| HN-C-V1 | 2.880E-03 | 0.966 | | 1.510E-03 | 0.973 | 1.230E-03 | 0.961 |
| HN-C-V2 | 2.740E-03 | 0.966 | | 1.470E-03 | 0.976 | 1.160E-03 | 0.962 |
| HN-C-V3 | 2.790E-03 | 0.967 | | 1.430E-03 | 0.970 | 1.210E-03 | 0.962 |
| HN-F-SM1 | 2.720E-03 | 0.986 | | 1.900E-03 | 0.917 | 8.182E-04 | 0.948 |
| HN-F-SM2 | 2.760E-03 | 0.986 | | 1.810E-03 | 0.908 | 8.302E-04 | 0.951 |
| HN-F-SM3 | 2.740E-03 | 0.986 | | 1.830E-03 | 0.915 | 8.058E-04 | 0.953 |
| HN-F-DV1 | 3.010E-03 | 0.977 | | 1.600E-03 | 0.878 | 8.050E-04 | 0.952 |
| HN-F-DV2 | 3.030E-03 | 0.977 | | 1.640E-03 | 0.879 | 7.850E-04 | 0.944 |
| HN-F-DV3 | 2.930E-03 | 0.977 | | 1.590E-03 | 0.881 | 7.888E-04 | 0.948 |
| HN-F-V1 | 3.570E-03 | 0.981 | | 2.000E-03 | 0.959 | 9.046E-04 | 0.942 |
| HN-F-V2 | 3.220E-03 | 0.989 | | 1.950E-03 | 0.960 | 9.349E-04 | 0.944 |
| HN-F-V3 | 3.270E-03 | 0.987 | | 1.880E-03 | 0.961 | 9.431E-04 | 0.951 |
| LN-C-SM1 | 1.770E-03 | 0.977 | | 7.668E-04 | 0.945 | 4.946E-04 | 0.949 |
| LN-C-SM2 | 1.840E-03 | 0.979 | | 7.768E-04 | 0.942 | 5.146E-04 | 0.950 |
| LN-C-SM3 | 1.760E-03 | 0.975 | | 7.831E-04 | 0.944 | 4.959E-04 | 0.948 |
| LN-C-DV1 | 1.500E-03 | 0.966 | | 7.639E-04 | 0.932 | 4.870E-04 | 0.956 |
| LN-C-DV2 | 1.470E-03 | 0.968 | | 7.693E-04 | 0.930 | 4.990E-04 | 0.961 |
| LN-C-DV3 | 1.480E-03 | 0.973 | | 7.552E-04 | 0.931 | 4.876E-04 | 0.960 |
| LN-C-V1 | 1.430E-03 | 0.977 | | 9.155E-04 | 0.946 | 5.318E-04 | 0.957 |
| LN-C-V2 | 1.570E-03 | 0.974 | | 9.398E-04 | 0.947 | 5.265E-04 | 0.956 |
| LN-C-V3 | 1.520E-03 | 0.972 | | 9.224E-04 | 0.947 | 5.075E-04 | 0.947 |
| LN-F-SM1 | 1.190E-03 | 0.978 | | 7.385E-04 | 0.951 | 3.405E-04 | 0.949 |
| LN-F-SM2 | 1.170E-03 | 0.978 | | 7.290E-04 | 0.950 | 3.411E-04 | 0.947 |
| LN-F-SM3 | 1.180E-03 | 0.978 | | 7.312E-04 | 0.946 | 3.420E-04 | 0.950 |
| LN-F-DV1 | 9.643E-04 | 0.978 | | 7.484E-04 | 0.957 | 3.922E-04 | 0.945 |
| LN-F-DV2 | 9.812E-04 | 0.983 | | 7.757E-04 | 0.961 | 3.963E-04 | 0.933 |
| LN-F-DV3 | 9.505E-04 | 0.983 | | 7.470E-04 | 0.957 | 3.852E-04 | 0.947 |
| LN-F-V1 | 1.140E-03 | 0.986 | | 7.302E-04 | 0.963 | 4.077E-04 | 0.937 |
| LN-F-V2 | 1.150E-03 | 0.986 | | 7.049E-04 | 0.959 | 4.042E-04 | 0.938 |
| LN-F-V3 | 1.150E-03 | 0.985 | | 7.031E-04 | 0.950 | 4.072E-04 | 0.937 |

*C_cum_(t)* is the cumulative amount of C released at 365 days (mg CO_2_-C g^-1^ OC), *C_0_* is the pre-incubation SOC content (mg C g^-1^ OC), and *k* is the decay rate constant (day^-1^).

**Table S2**. Model parameters for CO_2_ release rate from exponential equations (*R = Be*

*^(kT)^*) based on a 30-day incubation period at 15, 25, and 35°C. HN-C/LN-C/HaN-C/HeN-C and HN-F/LN-F/HaN-F/HeN-F represent soils from cropland and forest in Hunan, Liaoning, Hainan, and Henan Provinces, China, respectively.

| **Groups** | ***B*** | ***K*** | ***Q_10_*** | ***R^2^*** |
| --- | --- | --- | --- | --- |
| HN-C-SM1 | 4.60 | 0.050 | 1.645 | 0.986 |
| HN-C-SM2 | 4.59 | 0.050 | 1.654 | 0.986 |
| HN-C-SM3 | 4.61 | 0.049 | 1.639 | 0.986 |
| HN-C-DV1 | 3.34 | 0.056 | 1.749 | 0.983 |
| HN-C-DV2 | 5.06 | 0.044 | 1.545 | 0.999 |
| HN-C-DV3 | 4.17 | 0.049 | 1.636 | 0.997 |
| HN-C-V1 | 4.73 | 0.043 | 1.540 | 0.948 |
| HN-C-V2 | 4.62 | 0.042 | 1.525 | 0.909 |
| HN-C-V3 | 4.49 | 0.041 | 1.508 | 0.858 |
| HN-F-SM1 | 3.45 | 0.070 | 2.012 | 0.988 |
| HN-F-SM2 | 3.42 | 0.070 | 2.014 | 0.990 |
| HN-F-SM3 | 3.38 | 0.070 | 2.016 | 0.993 |
| HN-F-DV1 | 4.94 | 0.060 | 1.815 | 0.999 |
| HN-F-DV2 | 5.03 | 0.058 | 1.791 | 0.999 |
| HN-F-DV3 | 4.86 | 0.061 | 1.839 | 0.999 |
| HN-F-V1 | 4.11 | 0.067 | 1.950 | 0.983 |
| HN-F-V2 | 3.99 | 0.067 | 1.946 | 0.988 |
| HN-F-V3 | 4.05 | 0.067 | 1.948 | 0.986 |
| LN-C-SM1 | 2.75 | 0.044 | 1.556 | 0.891 |
| LN-C-SM2 | 2.81 | 0.044 | 1.554 | 0.907 |
| LN-C-SM3 | 2.88 | 0.044 | 1.554 | 0.922 |
| LN-C-DV1 | 2.88 | 0.048 | 1.608 | 0.999 |
| LN-C-DV2 | 3.05 | 0.047 | 1.597 | 0.989 |
| LN-C-DV3 | 3.23 | 0.046 | 1.586 | 0.968 |
| LN-C-V1 | 5.31 | 0.020 | 1.219 | 0.902 |
| LN-C-V2 | 5.60 | 0.019 | 1.206 | 0.853 |
| LN-C-V3 | 5.89 | 0.018 | 1.192 | 0.798 |
| LN-F-SM1 | 3.40 | 0.073 | 2.067 | 1.000 |
| LN-F-SM2 | 3.43 | 0.073 | 2.067 | 1.000 |
| LN-F-SM3 | 3.36 | 0.073 | 2.067 | 1.000 |
| LN-F-DV1 | 6.27 | 0.052 | 1.680 | 0.997 |
| LN-F-DV2 | 6.17 | 0.052 | 1.687 | 0.996 |
| LN-F-DV3 | 6.06 | 0.053 | 1.694 | 0.995 |
| LN-F-V1 | 5.68 | 0.056 | 1.756 | 0.968 |
| LN-F-V2 | 5.43 | 0.057 | 1.761 | 0.970 |
| LN-F-V3 | 5.17 | 0.057 | 1.766 | 0.972 |
| HaN-C-SM1 | 1.32 | 0.058 | 1.781 | 0.994 |
| HaN-C-SM2 | 1.34 | 0.058 | 1.782 | 0.993 |
| HaN-C-SM3 | 1.45 | 0.056 | 1.758 | 1.000 |
| HaN-C-DV1 | 0.80 | 0.067 | 1.944 | 0.929 |
| HaN-C-DV2 | 0.76 | 0.071 | 2.028 | 0.965 |
| HaN-C-DV3 | 0.73 | 0.074 | 2.102 | 0.984 |
| HaN-C-V1 | 1.28 | 0.056 | 1.742 | 0.925 |
| HaN-C-V2 | 1.32 | 0.057 | 1.763 | 0.933 |
| HaN-C-V3 | 1.36 | 0.058 | 1.782 | 0.939 |
| HaN-F-SM1 | 0.23 | 0.113 | 3.093 | 1.000 |
| HaN-F-SM2 | 0.23 | 0.117 | 3.216 | 0.994 |
| HaN-F-SM3 | 0.23 | 0.120 | 3.330 | 0.980 |
| HaN-F-DV1 | 1.05 | 0.068 | 1.976 | 0.693 |
| HaN-F-DV2 | 1.03 | 0.070 | 2.016 | 0.659 |
| HaN-F-DV3 | 0.93 | 0.076 | 2.147 | 0.751 |
| HaN-F-V1 | 0.56 | 0.081 | 2.239 | 0.991 |
| HaN-F-V2 | 0.54 | 0.084 | 2.321 | 0.988 |
| HaN-F-V3 | 0.52 | 0.087 | 2.394 | 0.985 |
| HeN-C-SM1 | 1.72 | 0.051 | 1.664 | 0.906 |
| HeN-C-SM2 | 1.77 | 0.053 | 1.690 | 0.808 |
| HeN-C-SM3 | 1.81 | 0.053 | 1.694 | 0.767 |
| HeN-C-DV1 | 3.17 | 0.035 | 1.412 | 0.858 |
| HeN-C-DV2 | 3.61 | 0.032 | 1.373 | 0.790 |
| HeN-C-DV3 | 4.07 | 0.028 | 1.326 | 0.761 |
| HeN-C-V1 | 3.58 | 0.024 | 1.270 | 0.985 |
| HeN-C-V2 | 3.63 | 0.025 | 1.288 | 0.100 |
| HeN-C-V3 | 3.82 | 0.026 | 1.290 | 0.998 |
| HeN-F-SM1 | 2.13 | 0.024 | 1.275 | 0.985 |
| HeN-F-SM2 | 2.43 | 0.022 | 1.246 | 0.859 |
| HeN-F-SM3 | 2.65 | 0.020 | 1.220 | 0.892 |
| HeN-F-DV1 | 2.66 | 0.025 | 1.280 | 0.954 |
| HeN-F-DV2 | 2.96 | 0.023 | 1.254 | 0.955 |
| HeN-F-DV3 | 3.11 | 0.022 | 1.249 | 0.972 |
| HeN-F-V1 | 3.15 | 0.021 | 1.232 | 0.963 |
| HeN-F-V2 | 3.46 | 0.018 | 1.202 | 0.900 |
| HeN-F-V3 | 3.81 | 0.016 | 1.168 | 0.803 |

*R* is the CO_2_ release rate at a given temperature in μg CO_2_-C g^-1^ OC day^-1^, and *T* is the incubation temperature (°C). *B* and *k* are fitted parameters.

**Table S3**. The longitude, latitude, MAT, MAP, and basic soil properties of the sampling sites.

| **Sampling sites** | **Ecosystem types** | **Longitude (°E)** | **Latitude (°N)** | **MAT (℃)** | **MAP (mm)** | **pH** | **TC (mg/kg)** | **TN (mg/kg)** | **DOC (mg/kg)** |
| --- | --- | --- | --- | --- | --- | --- | --- | --- | --- |
| HN-F | Natural forests | 111.31167 | 28.69170 | 17.1 | 1336 | 4.57 ± 0.01 | 10.39 ± 0.24 | 0.95 ± 0.02 | 21.41 ± 4.79 |
| LN-F | Natural forests | 124.14520 | 41.81850 | 7.4 | 747 | 4.60 ± 0.04 | 29.15 ± 3.48 | 1.49 ± 0.19 | 26.51 ± 0.73 |
| HAN-F | Natural forests | 109.12999 | 19.08257 | 21.4 | 1484 | 5.58 ± 0.07 | 17.70 ± 0.89 | 1.19 ± 0.08 | 13.83 ± 2.22 |
| HEN-F | Natural forests | 114.27833 | 31.96778 | 15.2 | 1105 | 4.33 ± 0.01 | 21.96 ± 0.30 | 1.57 ± 0.04 | 14.42 ± 3.11 |
| HN-C | Croplands | 111.27065 | 28.74871 | 17.2 | 1325 | 5.26 ± 0.29 | 13.30 ± 0.11 | 1.29 ± 0.01 | 55.40 ± 1.55 |
| LN-C | Croplands | 124.17580 | 41.80410 | 7.0 | 759 | 4.64 ± 0.03 | 7.58 ± 0.48 | 0.60 ± 0.06 | 14.47 ± 2.33 |
| HAN-C | Croplands | 109.02416 | 19.38924 | 24.6 | 1322 | 4.87 ± 0.10 | 7.51 ± 0.33 | 0.43 ± 0.05 | 22.78 ± 0.35 |
| HEN-C | Croplands | 114.50361 | 32.23389 | 15.5 | 1045 | 5.01 ± 0.09 | 6.98 ± 0.24 | 0.58 ± 0.01 | 10.10 ± 1.09 |

The basic soil properties are presented as mean ± std dev (n = 3) for each group. MAT, mean annual temperature; MAP, mean annual precipitation; TC, total carbon; TN, total nitrogen; DOC, dissolved organic carbon.

# References

1.	Tong D, Wang Y, Yu H *et al.* Viral lysing can alleviate microbial nutrient limitations and accumulate recalcitrant dissolved organic matter components in soil. *ISME J* 2023; **17**: 1247-1256.

2.	Albright MBN, Gallegos-Graves LV, Feeser KL *et al.* Experimental evidence for the impact of soil viruses on carbon cycling during surface plant litter decomposition. *ISME Commun* 2022; **2**: 24*.*

3.	Wang S, Yu S, Zhao X *et al.* Experimental evidence for the impact of phages on mineralization of soil-derived dissolved organic matter under different temperature regimes. *Sci Total Environ* 2022; **846**: 157517.

4.	Knight CG, Nicolitch O, Griffiths RI *et al.* Soil microbiomes show consistent and predictable responses to extreme events. *Nature* 2024; **636**: 690-696.

5.	Padfield D, Castledine M, Buckling A. Temperature-dependent changes to host-parasite interactions alter the thermal performance of a bacterial host. *ISME J*. 2020; **14**: 389-398.

6.	Demory D, Arsenieff L, Simon N *et al.* Temperature is a key factor in *Micromonas*-virus interactions. *ISME J*. 2017; **11**: 601-612.

7.	Garcia FC, Clegg T, O'Neill DB *et al.* The temperature dependence of microbial community respiration is amplified by changes in species interactions. *Nat Microbiol* 2023; **8**: 272-283.

8.	Zhang L, Song L, Wang B *et al.* Co-effects of salinity and moisture on CO_2_ and N_2_O emissions of laboratory-incubated salt-affected soils from different vegetation types. *Geoderma* 2018; **332**: 109-120.

9.	Qin S, Kou D, Mao C *et al.* Temperature sensitivity of permafrost carbon release mediated by mineral and microbial properties. *Sci Adv* 2021; **7**: eabe3596.

10.	Gentsch N, Wild B, Mikutta R *et al.* Temperature response of permafrost soil carbon is attenuated by mineral protection. *Glob Change Biol* 2018; **24**: 3401-3415.

11.	Li H, Yang S, Semenov MV *et al.* Temperature sensitivity of SOM decomposition is linked with a K-selected microbial community. *Glob Change Biol* 2021; **27**: 2763-2779.

12.	Yang X, Huang X, Cheng J *et al.* Diversity-triggered bottom-up trophic interactions impair key soil functions under lindane pollution stress. *Environ Pollut* 2022; **314**: 120293.

13.	Zhao H, Lin J, Wang X *et al.* Dynamics of soil microbial N-cycling strategies in response to cadmium stress. *Environ Sci Technol* 2021; **55**: 14305-14315.

14.	Huang Y, Dai Z, Lin J *et al.* Labile carbon facilitated phosphorus solubilization as regulated by bacterial and fungal communities in *Zea mays*. *Soil Biol Biochem* 2021; **163**: 108465.

15.	Spohn M, Klaus K, Wanek W *et al.* Microbial carbon use efficiency and biomass turnover times depending on soil depth - Implications for carbon cycling. *Soil Biol Biochem* 2016; **96**: 74-81.

16.	Brookes PC, Powlson DS, Jenkinson DS. Measurement of microbial biomass phosphorus in soil. *Soil Biol Biochem* 1982; **14**: 319-329.

17.	Ding X, Xu W, Li Z *et al.* Phosphate hinders the complexation of dissolved organic matter with copper in lake waters. *Environ Pollut* 2021; **276**: 116739.

18.	Shi W, Zhuang W, Hur J *et al.* Monitoring dissolved organic matter in wastewater and drinking water treatments using spectroscopic analysis and ultra-high resolution mass spectrometry. *Water Res* 2021; **188**: 116406.

19.	Huang M, Zhou M, Li Z *et al.* How do drying-wetting cycles influence availability of heavy metals in sediment? A perspective from DOM molecular composition. *Water Res* 2022; **220**: 118671.

20.	Ohno T. Fluorescence inner-filtering correction for determining the humification index of dissolved organic matter. *Environ Sci Technol* 2002; **36**: 742-746.

21.	Cheng Z, Shi J, He Y *et al.* Assembly of root-associated bacterial community in cadmium contaminated soil following five-year consecutive application of soil amendments: Evidences for improved soil health. *J Hazard Mater* 2022; **426**: 128095.

22.	Wei X, Ge T, Wu C *et al.* T4-like phages reveal the potential role of viruses in soil organic matter mineralization. *Environ Sci Technol* 2021; **55**: 6440-6448.

23.	Xu Z, Sun R, He T *et al.* Disentangling the impact of straw incorporation on soil microbial communities: Enhanced network complexity and ecological stochasticity. *Sci Total Environ* 2023; **863**: 160918.

24.	Tong D, Xiao H, Li Z *et al.* Stand ages adjust fluctuating patterns of soil respiration and decrease temperature sensitivity after revegetation. *Soil Sci Soc Am J*. 2020; **84**: 760-774.

25.	Bolyen E, Rideout JR, Dillon MR *et al.* Reproducible, interactive, scalable and extensible microbiome data science using QIIME 2. *Nat Biotechnol* 2019; **37**: 852-857.

26.	Callahan BJ, McMurdie PJ, Rosen MJ *et al.* DADA2: High-resolution sample inference from Illumina amplicon data. *Nat Meth* 2016; **13**: 581-583.

27.	Quast C, Pruesse E, Yilmaz P *et al.* The SILVA ribosomal RNA gene database project: improved data processing and web-based tools. *Nucleic Acids Res* 2013; **41**: D590-D596.

28.	Zheng B, Zhu Y, Sardans J *et al.* QMEC: A tool for high-throughput quantitative assessment of microbial functional potential in C, N, P, and S biogeochemical cycling. *Sci China Life Sci* 2018; **61**: 1451-1462.

29.	Bolger AM, Lohse M, Usadel B. Trimmomatic: a flexible trimmer for Illumina sequence data. *Bioinformatics* 2014; **30**: 2114-2120.

30.	Jung Y, Han D. BWA-MEME: BWA-MEM emulated with a machine learning approach. *Bioinformatics* 2022; **38**: 2404-2413.

31.	Li D, Liu C-M, Luo R *et al.* MEGAHIT: an ultra-fast single-node solution for large and complex metagenomics assembly via succinct *de* *Bruijn* graph. *Bioinformatics* 2015; **31**(10): 1674-1676.

32.	Ounit R, Wanamaker S, Close TJ *et al.* CLARK: fast and accurate classification of metagenomic and genomic sequences using discriminative *k*-mers. *BMC Genomics* 2015; **16**: 236.

33.	Parks DH, Chuvochina M, Rinke C *et al.* GTDB: an ongoing census of bacterial and archaeal diversity through a phylogenetically consistent, rank normalized and complete genome-based taxonomy. *Nucleic Acids Res* 2022; **50**: D785-D794.

34.	Parks DH, Imelfort M, Skennerton CT *et al.* CheckM: assessing the quality of microbial genomes recovered from isolates, single cells, and metagenomes. *Genome Res* 2015; **25**: 1043-1055.

35.	Woodcroft BJ, Singleton CM, Boyd JA *et al.* Genome-centric view of carbon processing in thawing permafrost. *Nature* 2018; **560**: 49-54.

36.	Chen SF, Zhou YQ, Chen YR *et al.* fastp: an ultra-fast all-in-one FASTQ preprocessor. *Bioinformatics* 2018; **34**: 884-890.

37.	Kopylova E, Noé L, Touzet H. SortMeRNA: fast and accurate filtering of ribosomal RNAs in metatranscriptomic data. *Bioinformatics* 2012; **28**: 3211-3217.

38.	Li DH, Liu CM, Luo RB *et al.* MEGAHIT: an ultra-fast single-node solution for large and complex metagenomics assembly via succinct de Bruijn graph. *Bioinformatics* 2015; **31**: 1674-1676.

39.	Buchfink B, Xie C, Huson DH. Fast and sensitive protein alignment using DIAMOND. *Nat Meth* 2015; **12**: 59-60.

40.	Dixon P. VEGAN, a package of R functions for community ecology. *J Veg Sci*. 2003; **14**: 927-930.

41.	Zhou X, Hao Y, Gu B *et al.* Microbial communities associated with methylmercury degradation in paddy soils. *Environ Sci Technol* 2020; **54**: 7952-7960.

42.	Delgado-Baquerizo M, Reith F, Dennis PG *et al.* Ecological drivers of soil microbial diversity and soil biological networks in the Southern Hemisphere. *Ecology* 2018; **99**: 583-596.

43.	Wei X, Ge T, Wu C *et al.* T4-like phages reveal the potential role of viruses in soil organic matter mineralization. *Environ Sci Technol* 2021; **55**: 6440-6448.

44.	Braga LPP, Spor A, Kot W *et al.* Impact of phages on soil bacterial communities and nitrogen availability under different assembly scenarios. *Microbiome* 2020; **8**: 52.

45.	Shelford EJ, Suttle CA. Virus-mediated transfer of nitrogen from heterotrophic bacteria to phytoplankton. *Biogeosciences* 2018; **15**: 809-819.

46.	Kuzyakov Y, Mason-Jones K. Viruses in soil: Nano-scale undead drivers of microbial life, biogeochemical turnover and ecosystem functions. *Soil Biol Biochem* 2018; **127**: 305-317.

47.	Jover LF, Effler TC, Buchan A *et al.* The elemental composition of virus particles: Implications for marine biogeochemical cycles. *Nat Rev Microbiol* 2014; **12**: 519-528.

48.	Feng J, Tang M, Zhu B. Soil priming effect and its responses to nutrient addition along a tropical forest elevation gradient. *Glob Change Biol* 2021; **27**: 2793-2806.

49.	Fang Y, Nazaries L, Singh BK *et al.* Microbial mechanisms of carbon priming effects revealed during the interaction of crop residue and nutrient inputs in contrasting soils. *Glob Change Biol* 2018; **24**: 2775-2790.

50.	Cui Y, Wang X, Zhang X *et al.* Soil moisture mediates microbial carbon and phosphorus metabolism during vegetation succession in a semiarid region. *Soil Biol Biochem* 2020; **147**: 107814.

51. Wang Y, Theodore M, Xing Z *et al*. Structural mechanisms of Tad pilus assembly and its interaction with an RNA virus. *Sci Adv* 2024; **10**: eadl4450.

52. Mekler V, Minakhin L, Sheppard C *et al*. Molecular mechanism of transcription inhibition by phage T7 gp2 protein. *J Mol Biol* 2011; **413**: 1016-1027.

53. Erb TJ, Berg IA, Brecht V et al. Synthesis Of C5-dicarboxylic acids from C2-units involving crotonyl-CoA carboxylase/reductase: The ethylmalonyl-CoA pathway. *Proc Natl Acad Sci U S A* 2007; **104**: 10631-10636.

54. Bingham R, Ekunwe SIN, Falk S et al. The major head protein of bacteriophage T4 binds specifically to elongation factor Tu. *J Biol Chem* 2000; **275**: 23219-23226.

55. Bell GS, Russell RJM, Connaris H *et al*. Stepwise adaptations of citrate synthase to survival at life's extremes - from psychrophile to hyperthermophile. Eur J Biochem. 2002; **269**: 6250-6260.
